# Muscle Abnormalities in Nonhospitalised Patients With Post–COVID‐19 Condition

**DOI:** 10.1002/jcsm.70085

**Published:** 2025-10-01

**Authors:** Andrea Tryfonos, Gustav Jörnåker, Håkan Rundqvist, Kaveh Pourhamidi, Michael Melin, Helena Wallin, Filip J. Larsen, Spyridon Pantelios, Anders P. Mutvei, Veronika Tillander, Uwe J. F. Tietge, Sergio Perez Diaz, Douglas Crafoord, Alen Lovric, Rodrigo Fernandez‐Gonzalo, Eric Rullman, Per Stål, Thomas Gustafsson, Helene Rundqvist, Tommy R. Lundberg

**Affiliations:** ^1^ Department of Laboratory Medicine, Division of Clinical Physiology Karolinska Institutet Huddinge Sweden; ^2^ Department of Life Sciences School of Sciences, European University Cyprus Nicosia Cyprus; ^3^ Unit of Clinical Physiology Karolinska University Hospital Huddinge Sweden; ^4^ Department of Clinical Neurophysiology Karolinska University Hospital Huddinge Sweden; ^5^ Division of Cardiology, Department of Medicine Karolinska Institutet Huddinge Sweden; ^6^ Department of Physiology, Nutrition and Biomechanics Swedish School of Sport and Health Sciences, GIH Stockholm Sweden; ^7^ Department of Laboratory Medicine, Division of Pathology Karolinska Institutet Huddinge Sweden; ^8^ Department of Laboratory Medicine, Division of Clinical Chemistry Karolinska Institutet Huddinge Sweden; ^9^ Clinical Chemistry, Karolinska University Laboratory Karolinska University Hospital Stockholm Sweden; ^10^ Department of Medical and Translational Biology Umeå University Umeå Sweden

**Keywords:** muscle abnormalities, muscle histology, myopathies, post–COVID‐19 condition, RNA sequencing, skeletal muscle

## Abstract

**Background:**

Post‐COVID condition (PCC) affects ~10% of SARS‐CoV‐2–infected individuals and manifests as persistent symptoms such as fatigue, exercise intolerance and muscle weakness. This study aimed to assess the skeletal muscle of these patients and compare them with healthy controls.

**Methods:**

Biopsies were obtained from the vastus lateralis muscle of 28 nonhospitalised PCC patients without concomitant diseases (75% women, mean age 46.4 ± 10.4 years) and 28 age‐ and sex‐matched healthy controls (79% women, mean age 46.6 ± 8.7 years). The analysis included morphological and pathological alterations, fibre type composition, fibre cross‐sectional area, capillarisation, number of myonuclei, presence of developmental myosin, CD68^+^ cells, macroautophagy markers, mitochondrial respiration, lipidomics and RNA sequencing.

**Results:**

PCC patients, compared to controls, had a higher percentage of angulated fibres (median [IQR] 0.43 [0.00–3.20] vs. 0.00 [0.00–0.00]; *p* < 0.001), small, rounded fibres (0.21 [0.00–1.20] vs. 0.00 [0.00–0.00]; *p* < 0.001) and fibres expressing fetal myosin (0.26 [0.00–1.15] vs. 0.00 [0.00–0.17]; *p* = 0.015). Semiquantitative analysis showed nuclear clumps (18/27, 66.6%), hypertrophic fibres (9/27, 33.3%) and fibrosis (22/27, 81.4%) in PCC patients. Fibre cross‐sectional area was significantly lower in PCC patients (4031 ± 1365 vs. 4982 ± 1463 μm^2^; *p* = 0.018), largely driven by differences in type 2 fibre size (3533 ± 1249 vs. 4275 ± 1646 μm^2^; *p* = 0.068) than type 1 fibre size (4553 ± 1422 vs. 4932 ± 1380 μm^2^; *p* = 0.325). There was a significantly lower number of myonuclei per fibre in PCC (3.4 ± 1.1 vs. 4.1 ± 1.0; *p* = 0.012), but no difference in the presence of CD68^+^ per fibre (0.28 ± 0.15 vs. 0.22 ± 1.0; *p* = 0.115). No group differences were observed in macroautophagy markers LC3B (0.0032 ± 0.0007 vs. 0.0030 ± 0.0006; *p* = 0.232) or p62 (0.0072 ± 0.0023 vs. 0.0079 ± 0.0016; *p* = 0.814). Capillary‐to‐fibre ratio in PCC patients was lower for both type 1 (2.2 ± 0.7 vs. 2.6 ± 0.9; *p* = 0.044) and type 2 fibres (1.8 ± 0.6, vs. 2.2 ± 0.8; *p* = 0.022). Mitochondrial respiration was 11–28% lower in PCC patients, although not statistically significant. Lipidomics showed a lower number of phospholipids, and RNA sequencing revealed downregulation of eight metabolic pathways, primarily related to oxidative phosphorylation in PCC patients compared to controls (FDR < 0.05).

**Conclusions:**

Nonhospitalised patients with PCC show signs of morphological and pathological muscle changes suggestive of degeneration and regeneration. The smaller overall fibre size, lower number of phospholipids, reduced mitochondrial oxidative capacity and lower capillarisation in these patients may be a consequence of reduced physical activity levels. The presence of clusters of atrophied angular and round‐shaped fibres, signs of inflammation and fibrosis and increased expression of fetal myosin may reflect myopathic and neurogenic post‐viral effects.

**Trial Registration:**

ClinicalTrials.gov Identifier: NCT05445830.

## 1 Introduction

Approximately 10% of people infected with the SARS‐CoV‐2 virus experience persistent symptoms more than 3 months after the initial infection [1, 2, 3], a condition known as post‐COVID condition (PCC). Common symptoms include fatigue, brain fog, shortness of breath and muscle pain, with a characteristic feature being postexertional malaise (PEM), in which physical exertion exacerbates symptoms [2]. Notably, a recent study reported that muscle pain, fatigue and PEM persist in patients even at a 3‐year follow‐up [4]. This, and the fact that even young individuals with a mild acute course of infection may experience PCC [5], raises concerns about the potential public health and economic burden of the condition.

We have recently shown that nonhospitalised and previously healthy individuals suffering from PCC have normal heart and lung function and no signs of systemic inflammation but show significantly reduced peak oxygen uptake and lower muscle strength compared to age‐ and sex‐matched healthy controls [6, 7]. Interestingly, >60% of PCC patients showed signs of myopathy assessed by needle electromyography (EMG), compared to only 1 case in the control group, indicating possible muscle complications in individuals with PCC. The observation that muscle involvement may be a concern for PCC patients is supported by recent studies indicating mitochondrial dysfunction, reduced capillarisation and elevated immune cell infiltration in skeletal muscle of PCC patients compared to historical [8] or prospective controls [9, 10]. However, the heterogeneity of patients, even within studies (i.e., inclusion of hospitalized patients experiencing long‐term symptoms, but not specifically qualifying for PEM), makes it difficult to draw a conclusion on the main features leading to muscle abnormalities in PCC. One study reported a higher proportion of type 2 fibres in PCC patients [9], supporting the tendency for more glycolytic fatiguing properties of the skeletal muscle, while two studies found no difference in muscle fibre distribution between PCC patients and controls [8, 10]. These contradictory findings emphasize the need to better understand the underlying muscular changes in PCC.

Given the diverse muscle alterations observed in PCC, including myopathic signs on needle EMG, we reasoned that greater attention should be paid to high‐quality analysis of skeletal muscle characteristics in this cohort. RNA sequencing offers powerful high‐throughput insights into the global gene expression and regulatory pathways, enabling the identification of differential expression of all genes in skeletal muscle [11]. Indeed, our previous work has emphasized the importance of the transcriptional response in explaining skeletal muscle responses to, for example, muscle atrophy induced by severe inactivity in healthy individuals [12]. Furthermore, lipidomics has increasingly revealed that reduced skeletal muscle function is associated with alterations in the muscle lipidome [13]. Applying these advanced techniques to PCC patients may provide insights into the potential molecular mechanisms underlying the skeletal muscle phenotype, which could help refine therapeutic targets and rehabilitation interventions.

To address this, we conducted a comprehensive analysis of muscle biopsies from our cohort, consisting of well‐defined nonhospitalised PCC patients and an equal number of age‐ and sex‐matched healthy controls, to further characterize the suspected muscle abnormalities. We performed morphological and pathological examinations, along with quantitative and qualitative immunochemical assessments of fibre type composition, cross‐sectional area, capillarisation, inflammation and macroautophagy markers. Additionally, we assessed mitochondrial respiration and performed targeted lipidomics and exploratory RNA sequencing to provide a comprehensive assessment of skeletal muscle properties. We hypothesised that PCC would be associated with a glycolytic shift and features of myopathy, including decreased mitochondrial function, supported by altered gene expression and pathway signatures.

## 2 Methods

### 2.1 Study Design

In the current study, we analyzed muscle biopsies from patients with PCC and an equal number of age‐ and sex‐matched healthy controls as part of a randomized controlled trial [6]. This prospective, randomized, controlled clinical trial (NCT05445830) was in accordance with the Declaration of Helsinki and was approved by the Swedish Ethical Review Authority (Identifiers: 2021‐05758‐01, 2022‐02999‐02). All participants provided written informed consent and underwent comprehensive physiological characterization at the units of clinical physiology and neurophysiology at Karolinska University Hospital. Details of the participants can be found elsewhere [6]. Briefly, patients were a) between 18 and 64 years of age, b) had laboratory‐confirmed COVID‐19 infection, c) persistent PEM symptoms for ≥ 3 months, verified by the DePaul Symptom Questionnaire, d) no hospitalization for COVID‐19, e) no history of cardiovascular/respiratory disease, generalized anxiety disorder, or somatic symptom disorder and f) no symptoms before March 2020.

### 2.2 Muscle Biopsies

After an overnight fast and no physical activity for 48 h, tissue samples were obtained from the vastus lateralis muscle under local anaesthesia using the percutaneous Bergström technique [14] with suction applied. The samples were quickly frozen in liquid nitrogen‐cooled isopentane and then stored at −80°C until further processing. In a subset of the cohort, a small portion of fresh muscle was used to analyze mitochondrial respiration, as described below.

### 2.3 Enzyme Histochemistry and Basic Histology

Frozen muscle samples were prepared for histological analysis. Briefly, muscle samples were cut into 7‐μm thick sections using a cryostat chamber (CryoStar NX70 HOMVPD, Thermo Fisher Scientific) and collected on glass slides (Superfrost Plus, Epredia) and stored at −80°C. The samples were stained with routine haematoxylin–eosin (H&E) [12] to show basic morphology, including degenerative processes. The mitochondrial enzyme nicotinamide adenine dinucleotide tetrazolium reductase (NADH‐TR) (EC1.6.99.3) was used to demonstrate myofibrillar oxidative activity and the density of mitochondria within the fibres.

### 2.4 Immunochemical Analysis

The muscle sections were processed for immunocytochemistry using previously characterized monoclonal antibodies (mAbs) (Table S1). Muscle fibre type identification was performed using a mAb against human slow‐contracting myosin heavy chain (MyHC) 1 (BA‐F8). The cell border of the muscle fibres and capillaries was identified with antibodies (Abs) directed against laminin, a major noncollagenous basement membrane component (basal lamina). DAPI (40,6‐diamidino‐2‐phenylindole) was used to determine myonuclear content. Monoclonal Ab 4C7 against laminin alpha‐5 chain strongly labels capillaries, mAb 5H2 against laminin alpha‐2 chain only labels the basement membrane of fibres, and polyclonal Ab 11 575 labels capillaries and muscle fibres. Developmental MyHC was identified with mAb NCL‐MHCn directed against fetal MyHC. Fibronectin, an extracellular matrix glycoprotein, was marked with a polyclonal antibody A0245. Macrophages were identified with mAb 68. Macroautophagy was marked with Ab LC3B and p62. Multilabeling was achieved by incubating the sections with a solution containing two or more mAbs.

In brief, before immunostaining with Abs BA‐F8, 11 575, 4C7, CD68, LC3B and p62, the muscle tissue sections were rehydrated in phosphate‐buffered saline (PBS) for 5 min and fixed in 4% paraformaldehyde for 10 min. After rinsing (in PBS containing 0.05% Tween) and permeabilization (in 0.5% Triton X‐100 in PBS), the sections were immersed in 5% normal goat serum (NGS or Carbo‐Free Blocking Buffer) for 30 min. The sections immunostained for Z0097, A0245 and 15200 were immersed in 5% normal nonimmune donkey serum (Jackson ImmunoResearch Europe Ltd., Cambridgeshire, Ely, UK) for 15 min and rinsed in 0.01 M PBS for 3 × 5 min. The samples were then incubated overnight at 4°C with the primary antibodies diluted to appropriate concentrations in NGS or bovine serum albumin (BSA). After overnight incubation, sections were rinsed in PBS, followed by a second block with 5% NGS or BSA for 30 min. After additional washes in PBS, bound primary Abs were visualised by indirect immunofluorescence using affinity‐purified secondary Abs prepared for multiple labelling (RRX) (Jackson ImmunoResearch Europe Ltd., Cambridgeshire, Ely, UK) and mounted in Prolong Gold Antifade reagent DAPI (Invitrogen by Thermofisher Scientific, Eugene, Oregon, USA) or fluorescence mounting medium (Agilent, S3023) (Table S1).

### 2.5 Morphological Analysis

For analysis of muscle fibre type, fibre size, myonuclear content and capillarisation, muscle cross‐sections were scanned and examined at 10× magnification using a Vectra 3.0 automated quantitative pathology imaging system (Akoya Biosciences. Inc) with the filter DAPI/FITC/Cy3/Cy5. Images were processed using CellProfiler software (Cellprofiler 4.2.6) and the pipeline “Muscle2View” to analyze muscle fibre characteristics, including fibre type composition, cross‐sectional area (CSA), myonuclear content and capillary metrics as previously described [13]. In addition, the coefficient of variation (CV%) of muscle fibre size was calculated. Macroautophagy markers (LC3B, p62) were analysed using **c**onfocal imaging (Nikon Confocal A1R+, Plan Apo λ 20× Ph2 DM objective (NA 0.75)). Image segmentation and analysis were performed in *ImageJ* (1.54f), ilastik 1.4 and CellProfiler 4.2. Tissue and border classifications were conducted via ilastik Pixel Classification workflow and Multicut algorithm for boundary‐based segmentation. Muscle fibre objects were identified via segmentation masks in CellProfiler. R script (R Studio software) was used to compile the CellProfiler output data for further statistical analysis. For analyses of muscle pathology and mitochondrial NADH‐TR activity, muscle cross‐sections were scanned and examined at 20x magnification using a microscope (Leica DM6000B, Leica Microsystems CMS GmbH, Wetzlar, Germany) equipped with a colour CCD camera (Leica DFC490) and a digital high‐speed fluorescence CCD camera (Leica DFC360 FX). The same investigator manually calculated the numbers of degenerative and regenerative fibres in each scanned section and the NADH‐TR activity in muscle fibres. All morphological analyses were performed blind to the different groups.

### 2.6 Mitochondrial Respiration

Mitochondria were isolated from fresh muscle tissue using previously described protocols [15, 16]. Muscle samples were cooled and homogenized in isolation medium treated with bacterial protease, and centrifuged to separate mitochondrial pellets, which were then resuspended for further analysis. Mitochondrial respiration was measured using a two‐channel Oroboros respirometer at 37°C, employing the MIR05 respiration medium (composition: 0.5 mM EGTA, 3 mM MgCl2·6H2O, 60 mM K‐lactobionate, 20 mM Taurine, 10 mM KH2PO4, 20 mM HEPES, 110 mM Sucrose, 1 g L^−1^ BSA). Calibration was performed according to the manufacturer's guidelines. Respiration was evaluated under various metabolic states using substrate combinations, including 0.2 mM octanoyl carnitine, 0.5 mM malate, 5 mM pyruvate, 2.5 mM ADP, 10 mM glutamate, 0.5 mM rotenone and 10 mM succinate. Protein content was quantified spectrophotometrically using the Pierce 660 nm protein assay. O_2_ consumption was normalized to the quantity of mitochondrial protein. Data acquisition and analysis were conducted using DatLab software.

### 2.7 Targeted Lipidomics

Lipids were extracted overnight from ~20 mg of weighed muscle tissue using Folch solution (1:2 methanol:chloroform) with internal standards (0.5 μg/mL). The organic phase was transferred to new glass tubes and dried under nitrogen. Lipids were reconstituted in one part hexane and two parts isopropanol to a final volume of 100 μL and transferred to vials for LC–MS/MS. Analysis was performed using a Waters Acquity UHPLC coupled to a Xevo TQ‐X mass spectrometer (Waters, Milford, MA, USA) with an ACQUITY C18 column (150 mm × 2.1 mm, 1.7 μm) at 60°C *. Mobile* phase A: 10 mM ammonium formate +0.1% formic acid in 60% acetonitrile; phase B: same additives in 10% acetonitrile/90% isopropanol (flow rate: 0.2 mL/min, gradient mode). Ionization was via UniSpray (2.0 kV), positive or negative mode by lipid class. Nitrogen was used as desolvation (1000 L/h) and cone gas (150 mL/min); argon was used as collision gas (0.15 mL/min). Cone voltage: 30 V (phospholipids, TAGs), 50 V (cholesteryl esters). Collision energy (16–24 eV) was optimized per lipid class. Quantification used six‐point calibration curves, with lipid standard/internal standard ratios, and data were expressed as w/v concentrations. Data were acquired and analysed using MassLynx (Waters).

### 2.8 RNA Extraction and RNA Sequencing

RNA was extracted from ~20‐mg frozen muscle samples. Briefly, a bead‐beater device (BioSpec Products Inc., Bartlesville, OK, USA) was used to homogenate the tissue in TriZol (Invitrogen Life Technologies, Carlsbad, CA, USA), followed by chloroform gradient separation and ethanol dilution. After this, the Direct‐zol RNA Miniprep Kit (Zymo Research, CA, USA) was used to purify the RNA following the manufacturer's specifications. The quality of RNA was assessed using the 2100 Bioanalyzer System, with RNA integrity number (RIN) ≥ 8.5 reported across all samples. We also quantified total RNA content in relation to muscle weight (ng/mg).

Poly‐A enriched libraries were pair‐end sequenced using NovaSeq6000 platform (Illumina) across six flow cells and seven lanes. The obtained raw sequencing reads were submitted to FastQC (v0.12.1) for quality control, and only samples with a mean sequence quality score (Phred) > 30 was further aligned to the reference (GRCh38) using Kallisto sequence pseudo‐aligner (v0.50.1). Of the average processed reads (~27 million/library), 70%–88% were aligned across all samples.

### 2.9 Statistics

Analysis was performed using IBM SPSS, version 29.0 (IBM Corp), GraphPad Prism 9 for lipidomics and R version 4.3.3 (R Foundation for Statistical Computing, Vienna, Austria) for gene‐expression analyses. Details of the power calculation, which was based on primary outcomes, have been published previously [6]. Continuous variables were compared using 2‐tailed Student *t* tests or nonparametric tests (Mann–Whitney *U* test) when appropriate, and χ^2^ tests were used to compare categorical variables between patients with PCC and controls. Results are presented as means (SDs) or medians (IQRs). Statistical significance was defined as two‐sided *p* < 0.05. False discovery rate (FDR) correction for lipidomics data was done using the Benjamini, Krieger, Yekutieli method. Gene‐expression analyses were performed with R version 4.3.3 (R Foundation for Statistical Computing, Vienna, Austria). For gene‐expression analyses, the estimated transcript‐level abundances were imported and collapsed to the gene‐level using tximport (v1.26.1), ensuring appropriate correction for the average transcript length across the samples. Trimmed mean M normalization, conversion to log2CPM (counts per million) values, and subsequent differential gene expression analysis were conducted using edgeR (v3.40.2) [17]. Gene set enrichment analysis was performed with clusterProfiler (v4.15.1) [18] with pathways annotations from WikiPathways [17].

## 3 Results

Out of 62 subjects enrolled in this study (PCC, *n* = 31, controls, n = 31) [6], muscle biopsies were available from 56 participants (PCC, *n* = 28; controls, n = 28; 1 subject dropped out following baseline visit, for two subjects, technical issues on the experimental day prevented biopsies, and 1 subject was excluded retrospectively due to suspected rheumatoid arthritis). For histology and immunochemistry, frozen muscle was analyzed from 54 subjects (PCC, *n* = 27; controls, *n* = 27; we could not perform histological or immunochemical analysis on two subjects due to limited biopsy material for sectioning), while mitochondrial respiration experiments were performed in a subgroup of 10 patients with PCC and 11 age‐ and sex‐matched controls. Participants' characteristics are presented in Table 1.

**TABLE 1:** Characteristics for the post‐COVID condition patients (*n* = 28) and age‐ and sex‐matched healthy controls (*n* = 28).

	PCC patients	Controls	*p*
Age, mean (SD), years	46.4 (10.4)	46.6 (8.7)	0.934
Sex, *n* (%), women	21 (75%)	22 (79%)	0.752
BMI, mean (SD), m^2^/kg	25.2 (3.1)	25.4 (3.6)	0.818
Diabetes, *n* (%)	0 (0)	2 (7)	0.150
Hypertension, *n* (%)	4 (14)	4 (14)	1.000
Smoker, *n* (%)	1 (4)	2 (7)	0.553
Beta blocker, *n* (%) ^a^	7 (25)	2 (7)	0.069
COVID‐19 infection, *n* (%) ^b^	28 (100)	16 (57)	
COVID‐19 vaccinated, *n* (%)	25 (89)	26 (93)	0.639
Duration PCC symptoms	21.2 (9.5)	NA	
mMRC dyspnoea, mean (SD), score	1.9 (1.0)	0.6 (0.6)	*p* < 0.001
PCFS, mean (SD), score	2.6 (0.8)	NA	
Godin‐Sheperd Leisure Time Physical Activity Questionnaire			
Before COVID‐19, mean (SD), units	50.1 (19.1)	NA	
Current, mean (SD), units	23.7 (15.6)	50.6 (27.8)	*p* < 0.001
SF‐36 Questionnaire			
Physical function, mean (SD), score	50.4 (19.2)	92.0 (4.7)	<0.001
Physical role, mean (SD), score	11.6 (22.0)	95.5 (19.3)	<0.001
Emotional role, mean (SD), score	73.8 (41.9)	98.8 (6.3)	0.004
Mental health, mean (SD), score	23.8 (20.6)	75.4 (13.7)	<0.001
Social function, mean (SD), score	68.0 (19.5)	85.3 (10.2)	<0.001
Bodily pain, mean (SD), score	42.0 (26.2)	95.1 (13.3)	<0.001
Vitality, mean (SD), score	44.7 (26.0)	90.4 (15.9)	<0.001
General health, mean (SD), score	39.8 (18.1)	83.8 (10.3)	<0.001
Physical function			
6‐Min walk, mean (SD), m	457 (83)	490 (67)	0.102
Isometric 120 deg., mean (SD), N*m	149.2 (54.8)	186.5 (67.2)	0.027
Handgrip MVC, mean (SD), kg	36.4 (10.9)	40.9 (17.3)	0.250
Accelerometers			
MVPA, mean (SD), min/day	34.9 (28.8)	60.3 (31.2)	0.004
Total PA, mean (SD), min/day	317.1 (267.9)	299.6 (76.4)	0.755
Sedentary time, mean (SD), min/day	578.6 (78.1)	565.1 (72.4)	0.525
Blood biomarkers			
Leukocytes, mean (SD), x10(9)/L	5.6 (1.3)	5.7 (1.6)	0.836
Erythrocytes, mean (SD), x10(12)/L	4.4 (0.4)	4.4 (0.5)	0.845
Thrombocytes, mean (SD), x10(9)/L	251.7 (54.5)	259.5 (78.5)	0.681
Haemoglobin, mean (SD), g/L	132.8 (10.8)	132.2 (10.5)	0.839
CRP, mean (SD), mg/L	0.9 (1.0)	1.2 (1.4)	0.411
CPET			
VO_2 peak,_ mean (SD), ml/kg/min	29.2 (7.8)	35.5 (7.4)	0.003
Electromyography			
Normal, *n* (%)	10 (37)	24 (92)	
Myopathic indices, *n* (%)	12 (44)	0 (0)	
Myopathic indices borderline, *n* (%)	4 (15)	1 (4)	
Neuropathic indices, *n* (%)	1 (4)	1 (4)	<0.001

*Note:* Data are means and (SD) unless otherwise stated. Independent Student *t* test was used to assess the differences between the groups for continuous variables and χ^2^ for categorical variables. Significance was set to *p* < 0.05.

Abbreviations: BMI: body mass index; mMRC: modified medical research council scale for dyspnoea 0–4; PCFS: post‐COVID functional status 0–4; MVC: maximal voluntary contraction; PA: physical activity; MVPA: moderate and vigorous PA; VO_2_: volume of oxygen consumption.

^a^ Current use.

^b^ Verified COVID‐19 infection.

### 3.1 General Muscle Morphology and Pathology

**FIGURE 1:**
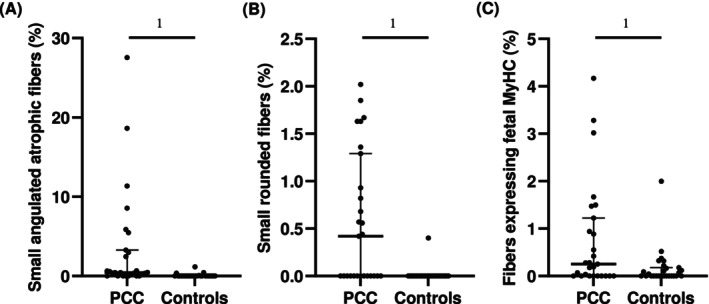
A) Presence of small angular atrophic fibres, B) presence of small, rounded fibres and C) fibres expressing fetal MyHC in the skeletal muscle of patients with PCC (*n* = 27) and age‐ and sex‐matched healthy controls (*n* = 27). Data are presented as median and IQR. Mann–Whitney test was used to assess the differences between the groups. 1 Significance was set to *p* < 0.05. IQR, interquartile range; MyHC, myosin heavy chain; PCC, post‐COVID condition.

**FIGURE 2:**
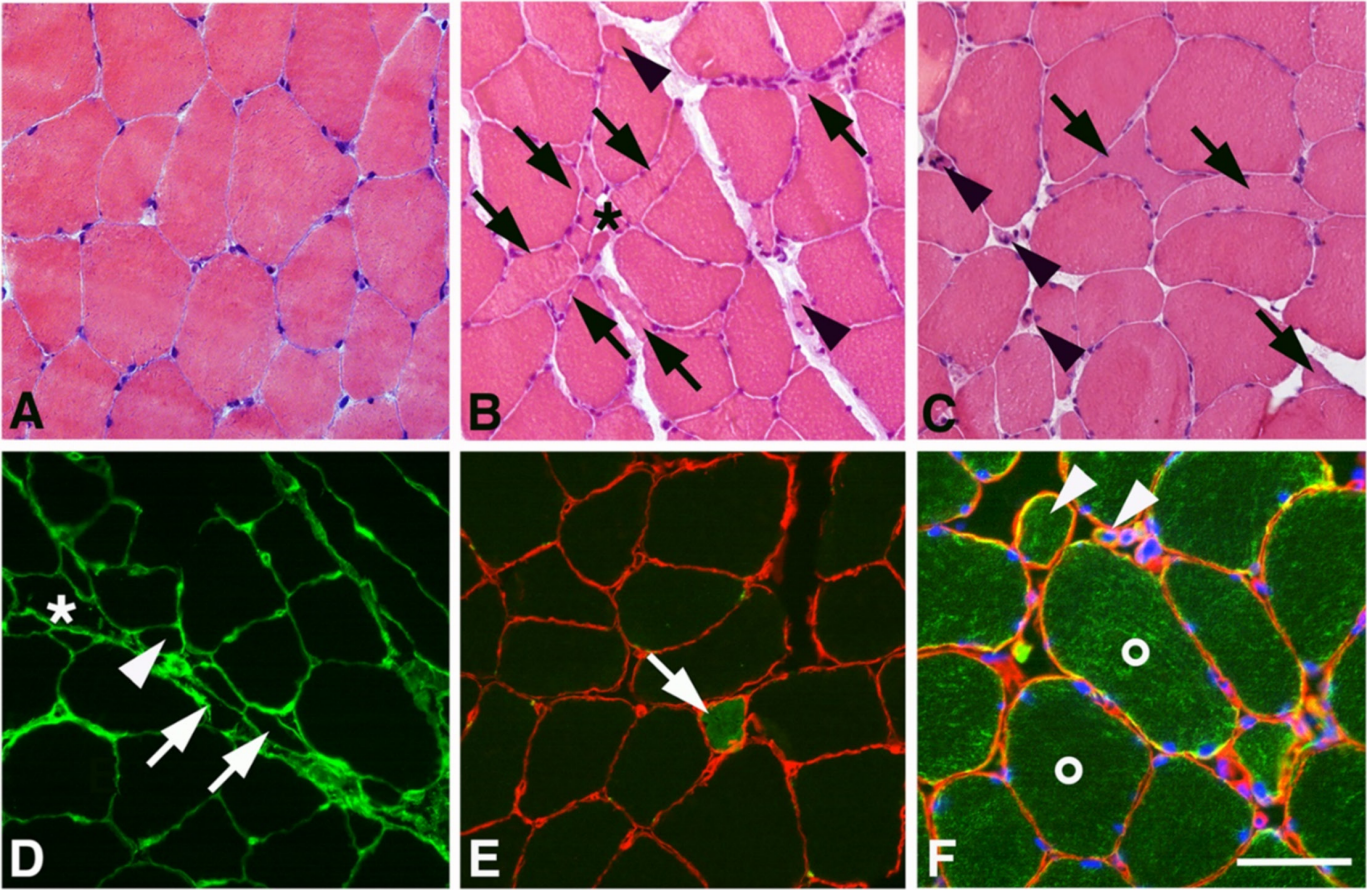
Muscle cross‐sections from a control subject (A) and PCC patients (B–F) histologically stained for H&E (A–C), immunohistochemically stained for fibronectin (D), double‐stained for laminin (red) and fetal MyHC (green) (E) and triple stained for desmin (green), laminin (red) and DAPI (blue) (F). Note the presence of atrophic angular fibres (arrows), sometimes clustered (asterisk) and nail‐shaped, and very small fibres with a more rounded appearance (arrowheads, B–D, F) in PCC patients. D illustrates a region with fibrosis and clusters (asterisks) of atrophic angulated (arrows) and round‐shaped fibres (arrowhead). A muscle fibre expressing fetal MyHC is shown in (E) (arrow), and a mixture of atrophic (arrowhead) and hypertrophic fibres (white circle) is illustrated in F. Bars = 100 μm.

**FIGURE 3:**
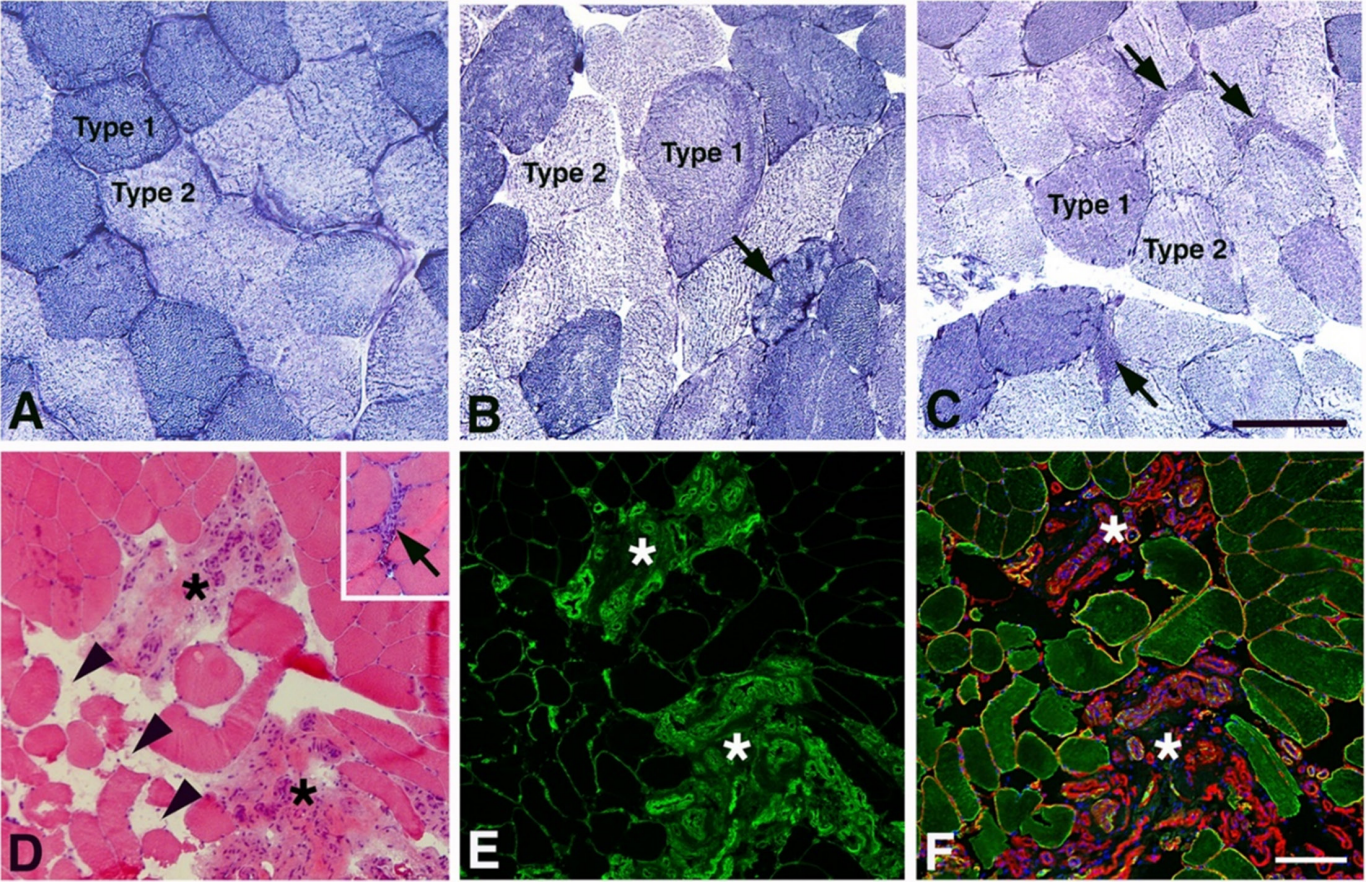
Muscle cross‐sections from a control subject (A) and PCC patients (BF) histochemically stained for mitochondrial NADH‐TR (A–C), H&E (D) and immunohistochemically for fibronectin (E), as well as triple stained for desmin (green), laminin (red) and DAPI (blue) (F). Note the generally lower mitochondrial staining for NADH‐TR, particularly in type 2 fibres of PCC patients (B, C) compared to controls (A). Observe also the fibre with an abnormal distribution and subsarcolemmal accumulation of mitochondria (arrow, B) and the characteristic high NADH‐TR staining activity in denervated atrophic angular fibres (arrows, C), regardless of whether they are type 1 or type 2 fibres. D–F shows an area with extensive infiltration of inflammatory cells and fibrosis (asterisks), fat infiltration (arrowheads) and ingrowth of vessels (red in F). Phagocytes invading necrotic muscle fibres can be seen in the areas marked with asterisks (D). The inset in D reveals a small area infiltrated by inflammatory cells in another PCC patient. Bars = 100 μm.

The muscle samples from PCC patients differed from the controls by having a significantly higher proportion of small‐sized fibres with an angular (*p* < 0.001, Figure 1A) or rounded form (*p* < 0.001, Figure 1B). The atrophic angular fibres appeared often in small clusters (Figure 2). Angular fibres were observed in 21/27 (77.7%) of PCC patients compared to 4/27 (14.8%) in controls, while small‐rounded fibres were found in 14/27 (51.8%) of PCC patients and 1/27 (3.0%) of controls (Figure 2). Nuclear clumps, the final stage of the fibres after denervation, were observed in 18 out of 27 PCC patients (66.6%), and clusters of hypertrophic fibres were found in 9 of the 27 patients (33.3%) (Table S2). Semiquantification of the sections stained for H&E and fibronectin showed fibrosis in 22 PCC patients (81.4%) (Figures 2, and 3, Table S2). A low amount of necrotic muscle fibres invaded by phagocytes was found in 17/27 (62.9%) PCC patients (Figure 3, Table S2). One PCC case revealed a local area in the muscle characterized by severe fibrosis, fat infiltration and an accumulation of inflammatory cells (Figure 3). All samples exhibited a normal staining pattern for muscle‐specific intermediate filament desmin and basement membrane laminin. Semiquantitative analysis revealed a generally lower mitochondrial NADH‐TR staining activity in PCC patients than in controls, particularly in type 2 fibres (Figure 3). Some PCC cases in the PCC group showed a few fibres where the pattern of the mitochondria was disorganized. No increased number of internal nuclei was observed. A significantly higher proportion of fibres expressing fetal MyHC was found in PCC patients compared to controls (*p* = 0.017) (Figures 1, 2). For an overview of abnormal and pathological findings, see Table S2.

### 3.2 Immunochemistry Analysis

**FIGURE 4:**
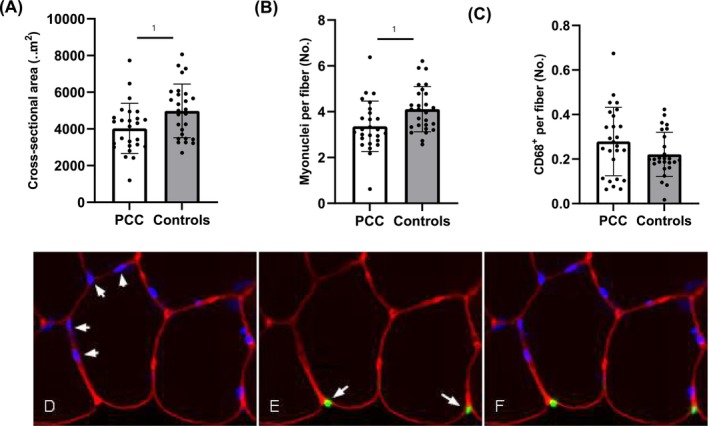
Average cross‐sectional area (A), myonuclei per fibre (B) and presence of CD68^+^ cells (C) in skeletal muscle of patients with post‐COVID condition (PCC; *n* = 27) and age‐ and sex‐matched controls (*n* = 27). A representative image from a PCC patient (D, E, F), histochemically stained for DAPI (blue) and laminin for borders (red), CD68^+^ (green) and laminin for borders (red). Data are presented as means and (SD). Independent Student *t* test was used to assess the differences between the groups. 1 Significance set to *p* < 0.05.

**FIGURE 5:**
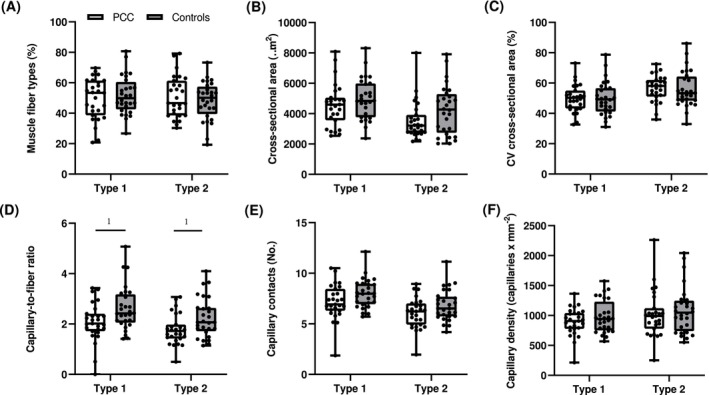
Muscle fibre characteristics for the PCC patients (*n* = 27) and age‐ and sex‐matched healthy controls (*n* = 27). Data are presented as means and (SD). Independent Student *t* test was used to assess the differences between the groups. 1 Significance was set to *p* < 0.05. PCC, post‐COVID condition.

Patients with PCC had lower average fibre CSA compared to controls (*p* = 0.018, Figure 4A). There was a tendency of lower CSA, particularly in type 2 fibres (~17%, *p* = 0.068), but not in type 1 fibres (*p* = 0.325) in patients with PCC compared to controls (Figure 5B). CV% in fibre size was similar between the groups for both fibre types (*p* > 0.05, Figure 5C). Fibre type specific analyses showed no difference in fibre type distribution between patients with PCC and controls (Figure 5A). There was a lower number of myonuclei per fibre in PCC patients compared to controls (*p* = 0.012, Figure 4B). There was no difference in the presence of CD68^+^ per fibre in skeletal muscle of PCC patients compared to controls (*p* = 0.115, Figure 4C), but there was a lower number of CD68^+^ per muscle area in PCC compared to controls (*p* = 0.004, Table S3). Likewise, no difference was observed in myofiber LC3B or p62 intensity (average myofiber intensity per subject; *p* = 0.232, *p* = 0.814, Figure S1). Capillary‐to‐fibre ratio was lower in both type 1 (~16%, *p* = 0.044) and type 2 fibres (~20%, *p* = 0.024) in patients with PCC compared to controls (Figure 5D). Overall, there was a tendency of a lower number of capillaries irrigating fibres (type 1, *p* = 0.077; type 2, *p* = 0.080, Figure 5E), as well as capillary‐to‐fibre perimeter exchange index (type 1, *p* = 0.052, type 2, *p* = 0.073) in PCC patients compared to controls, whereas supplying capillaries per area did not differ between the groups (type 1, *p* = 0.212, type 2, *p* = 0.659) (Figure 5F).

### 3.3 Mitochondrial Respiration

Mitochondrial respiration was assessed in a subgroup of the cohort (10 patients with PCC and 11 age‐ and sex‐matched controls. Separate statistical tests confirmed sex‐ and age‐matched subgroups; *p* > 0.05). Although mitochondrial quality (electron‐transferring flavoprotein complex Leak; ETF_L_, electron‐transferring flavoprotein complex oxidative phosphorylation; EFT_P_, complex I oxidative phosphorylation; CI_P_, complex I + II–linked substrate state oxidative phosphorylation; CI + II_P_) was numerically lower (~11%–28%) in the PCC group compared to controls, there were no statistical differences between the groups (Table 2).

**TABLE 2:** Mass‐specific mitochondria.

	PCC patients	Controls	*p*
EFT_L,_ mean (SD), per μg mitochondria protein	0.45 (0.29)	0.63 (0.36)	0.231
EFT_P,_ mean (SD), per μg mitochondria protein	2.73 (1.98)	3.07 (1.71)	0.698
CI_P,_ mean (SD), per μg mitochondria protein	4.92 (3.19)	6.54 (5.95)	0.453
CI + II_P,_ mean (SD), per μg mitochondria protein	9.62 (4.87)	10.82 (8.51)	0.699

*Note:* Respiration in a subgroup of 10 PCC patients and 11 age‐ and sex‐matched healthy controls. Data are presented as means and (SD). Independent Student *t* test was used to assess the differences between the groups. Significance was set to *p* < 0.05.

Abbreviations: O_2_ flux is expressed in pmol × s^−1^ × μg protein. Electron‐transferring flavoprotein complex (ETF), complex I (CI), complex I + II‐linked substrate state (CI + II). LEAK respiration and OXPHOS are indicated by subscripts L, P.

### 3.4 Targeted Lipidomics

Out of 650 lipid species included in the lipidomics panel, 350 were reproducibly detected across all skeletal muscle samples (Table S4). There were no lipids that were exclusively present in either group. After statistical correction for multiple testing, particularly phospholipid species were significantly lower in PCC patients.

### 3.5 RNA Content and Expression

Total RNA content was similar between groups (mean [SD] for PCC patients: 110.1 [43.0] ng/mg, controls: 94.7 (38.9) ng/mg; *p* = 0.159).

RNA‐sequencing analysis revealed that 11 genes were differently expressed in PCC patients compared with controls (false discovery rate [FDR] < 0.05), out of which five had a significant fold change (logFC ≥ 0.5). In particular, three genes mainly related to mitochondrial function (NIPSNAP1, logFC = 0.55, FDR = 0.024), metabolite transport (SLC16A12, logFC = 0.51, FDR = 0.009) and endothelial function (PLVAP, logFC = 0.75, FDR = 0.024) were lower expressed in PCC patients compared to controls, whereas two genes related to basic cellular processes including DNA packaging (NAP1L4, logFC = −3.43, FDR = 3.19 × 10^−9^), and tissue repair (RNF122, logFC = −0.503, FDR = 0.024) were higher expressed in PCC patients compared to controls.

Gene‐set enrichment analysis identified 8 differentially regulated pathways between patients and controls (FDR < 0.05), all of which had a lower expression in PCC patients than in controls. All the differentially expressed pathways were involved in metabolism, and in particular, oxidative phosphorylation (Table 3).

**TABLE 3:** Gene‐set enrichment analysis in skeletal muscle of patients with PCC (*n* = 28) and controls (*n* = 28).

Pathway	Set size	Enrichment score	*p*	*p* (adjusted)
Electron transport chain (OXPHOS system in mitochondria)	71	0.84	1 × 10^−10^	7 × 10^−9^
Oxidative phosphorylation	37	0.84	1 × 10^−10^	7 × 10^−9^
Nonalcoholic fatty liver disease	80	0.67	1 × 10^−10^	7 × 10^−9^
Mitochondrial complex I assembly model OXPHOS system	33	0.81	5 × 10^−10^	3 × 10^−8^
Amino acid metabolism	44	0.70	3 × 10^−7^	1 × 10^−5^
Metabolic reprogramming in colon cancer	23	0.71	2 × 10^−4^	9 × 10^−3^
mRNA targets in ECM and membrane receptors	16	0.76	3 × 10^−4^	1 × 10^−2^
Glycolysis and gluconeogenesis	22	0.69	9 × 10^−4^	2 × 10^−2^
Cytoplasmic ribosomal proteins	58	0.51	1 × 10^−3^	3 × 10^−2^

*Note:* *p* value (adjusted) were calculated using the Benjamini–Hochberg method.

## 4 Discussion

This study shows that nonhospitalised and previously healthy PCC patients have pathological skeletal muscle characteristics including clusters of small angular fibres and a notably higher presence of small round fibres. Moreover, signs of inflammation, fibrosis and fatty infiltration were observed in some PCC cases, and patients had a higher frequency of fibres expressing fetal myosin, a lower number of myonuclei per fibre, a smaller muscle fibre cross‐sectional area, reduced capillarisation and reduced phospholipid species. In addition, RNA sequencing revealed lower gene expression of pathways involved in oxidative phosphorylation and glycolysis in patients with PCC.

Our morphological and electrophysiological findings indicate both neurogenic and myopathic involvement in the muscle tissue of PCC patients. The neurogenic component is evident in small clusters of atrophic angular fibres, as well as the presence of nuclear clumps and fetal MyHC, all of which indicate a scattered loss of motor units, characteristic of denervation [19, 20]. This is consistent with the increasing evidence linking SARS‐CoV‐2 to peripheral nervous system manifestations [21, 22], particularly motor neuropathy [23]. The mechanism may involve immune system dysregulation, which has been proposed as a link between SARS‐CoV‐2 and peripheral nervous system manifestations [21], possibly causing local nerve damage in some motor units.

The myopathic involvement is supported by our previous reports of myopathic indices on needle EMG in > 60% of PCC patients [6, 7], together with recent observations of inflammatory cell infiltration, fibrosis and local fat deposition [24]. However, consistent changes in local fat deposition that would be mirrored by neutral lipids, mainly triglycerides, could not be replicated in the current work using a broad targeted lipidomics panel. The degenerative process is further evidenced by a lower number of myonuclei per fibre and an increased number of small fibres expressing fetal MyHC, a marker linked to both muscle denervation and regeneration [20, 25, 26]. In addition, targeted lipidomics revealed significantly reduced phospholipids in PCC patients, suggesting fewer membrane structures. This is consistent with the signs of muscle atrophy and mitochondria impairments [27]. While we observed increased infiltration of CD68^+^ myeloid cells per fibre area, the total number of these cells per fibre was not significantly different from controls. This contrasts with previous studies reporting a higher presence of various immune cells in PCC patients [8, 9]. The role of inflammation remains unclear, with conflicting evidence of systemic inflammatory markers such as IL‐6 in different cohorts [6, 28, 29]. Although we observed myositis in one patient and mild signs in others, the temporal relationship between SARS‐CoV‐2 infection and muscle inflammation needs further investigation. The combination of fibrosis and fatty infiltration suggests an early inflammatory response, as these changes typically occur when muscle regeneration is impaired. Although the relatively low number of denervated fibres suggests limited functional effects, our results suggest that PCC patients exhibit both degenerative and regenerative muscle changes that likely reflect an adaptive response to reduced physical activity in combination with postviral myopathic and neurogenic effects.

The overall lower fibre CSA in PCC patients seemed to be driven primarily by type 2 fibres, which are particularly susceptible to disuse [30]. Although our PCC patients were neither hospitalized nor bedridden, they had reduced moderate‐to‐vigorous physical activity [6]. Aschman et al. [8] also reported selective atrophy of type 2 fibres in 72% (8/11) of patients with PCC compared to historical cohorts, which is consistent with the overall lower fibre CSA of PCC patients compared to controls previously reported [10]. We also found a lower number of myonuclei per fibre in patients with PCC compared to controls, a feature typically associated with muscle atrophy [31]. In contrast, Appelman et al. [9] reported lower fibre CSA of type 1 but not in type 2 fibres, and in women only. They also found a higher proportion of type 2 fibres in PCC patients compared to controls, primarily due to higher expression of type 2X myosin in the patient group, a feature that usually occurs in more severe muscle disuse and disease. Nonetheless, these collective findings support that PCC can lead to measurable changes in muscle fibre composition and morphology, particularly affecting the more metabolically active type 2 fibres.

The tendency for lower NADH‐TR activity in type 2 fibres in our PCC patients compared to controls suggests impaired mitochondrial oxidative capacity, which may contribute to impaired ATP production and thus premature glycolytic fibre fatigue and reduced power output [6]. In addition, impaired mitochondrial function may increase the production of reactive oxygen species that damage cellular components and exacerbate fibre degeneration, leading to muscle atrophy [32, 33]. Our measurements in isolated mitochondria show that mitochondrial respiration was approximately 20% lower in PCC patients compared to controls. Although we could not detect a statistically significant difference in mitochondrial respiration, probably due to reduced statistical power (10 PCC patients and 11 controls), these findings, complemented by the global gene expression data, indicated lower expression of gene clusters related to both mitochondrial function and glycolysis, which may reflect a broader metabolic disturbance in PCC patients. These findings are consistent with previous observations indicating decreased mitochondrial respiration [9, 10], lower protein expression of key molecular markers of mitochondrial function and biogenesis, including citrate synthase and peroxisome proliferator‐activated receptor‐γ coactivator (PGC)1α [10] and higher glycolytic metabolites in skeletal muscle of PCC patients compared to controls [9]. While these changes may perhaps be explained by the lower physical activity levels in the patient cohort, we cannot exclude the possibility of a postviral effect on skeletal muscle, which could then lead to reduced muscle function and exercise capacity. Although dysregulated macroautophagy, reported in SARS‐CoV‐2–infected lung tissue [34] and certain myopathies [35, 36], may also contribute to dysregulated mitochondrial homeostasis and cellular metabolism, we found no evidence of such changes through LC3B or p62 staining in PCC muscle tissue.

Another indication of metabolic changes was that PCC patients had a lower capillary‐to‐fibre ratio in both fibre types compared to controls, which is crucial for efficient oxygen and nutrient delivery to muscle fibres. These findings were consistent with lower capillary contacts and capillary‐to‐fibre perimeter exchange. A reduced capillary‐to‐fibre ratio can impair mitochondrial respiration, oxidative phosphorylation and muscle function, ultimately contributing to muscle atrophy, reduced strength and lower exercise capacity [37, 38, 39, 40]. Although the reduced ratio observed in our study likely primarily reflects an adaptation to the decreased fibre CSA and lower mitochondrial oxidative capacity in PCC patients, we cannot rule out the possibility of vascular endotheliitis following SARS‐CoV‐2 infection either directly via the angiotensin‐converting enzyme (ACE)2 receptor or as a consequence of a postviral immune response [41]. Of note, our PCC cohort had higher arterial stiffness compared to controls, indicating endothelial dysfunction [6]. Aschman et al. [8] also reported a lower capillary‐to‐fibre ratio in more severely affected PCC patients (including hospitalized patients) compared to historical biopsy cohorts, while Appelman et al. [9] found a trend (*p* > 0.08) towards a lower capillary‐to‐fibre ratio in nonhospitalised PCC patients compared to controls. Taken together, these observations suggest that the PCC‐related muscle phenotype consists of a combination of selective fibre atrophy, lower capacity for aerobic metabolism, oxidative stress and reduced vascular support, which likely contributes to the characteristic symptoms of fatigue and reduced exercise capacity and muscle strength.

While this study provides valuable insight into skeletal muscle changes in PCC patients, there are some limitations that should be acknowledged. The morphological analysis showed a large variability between subjects, which may reflect the relatively high random error associated with histochemical analysis of muscle biopsies [42] and the wide age range of participants in the study. This reduces the power to detect group differences. It is worth noting that the current muscle analysis is part of a larger randomized clinical trial, and the power analysis was performed for the primary outcomes of this study (symptoms exacerbation after exercise [6]). Nevertheless, our cohort consisted of well‐defined, nonhospitalised and previously healthy PCC patients who all met criteria for PCC and PEM, with the majority of patients having myopathic signs as measured by needle EMG [6]. In addition, another limitation of this study is the exclusive labelling of type 1 fibres, which limits the ability to detect co‐expression of MyHC isoforms (e.g., for type 2A and 2X). Despite probable atrophy of type 2 fibres and evidence of muscle abnormalities, patients showed no significant worsening of symptoms in response to acute strength training and had only a ~20% lower aerobic capacity and muscle strength compared with controls and were generally able to perform exercise [6]. We therefore urge caution in interpreting the clinical significance of these results and recommend follow‐up to monitor improvement over time. A rehabilitation programme that focuses on strength training may be recommended as it is likely to counteract type 2 fibre atrophy, improve muscle strength, and increase functional capacity without excessive energy demands.

## 5 Conclusions

Our results show that PCC is associated with peripheral impairment and suggest possible skeletal muscle involvement in nonhospitalised, previously healthy patients with persistent PEM symptoms and reduced exercise capacity and muscle strength. The analysis indicated overall muscle fibre atrophy, downregulation of genes related to reduced oxidative capacity, lower phospholipids and lower capillarisation. These effects may primarily be explained by the lower activity level in this cohort. In contrast, the histopathological changes, such as atrophic angular and round‐formed fibres, inflammation, fibrosis and increased presence of fibres expressing fetal myosin, are likely more specific consequences of the postviral state. Measures should be taken to cautiously improve physical activity in this cohort to counteract further muscle atrophy and functional decline.

## Ethics Statement

The authors of this manuscript certify that they comply with the ethical guidelines for authorship and publishing in the *Journal of Cachexia, Sarcopenia and Muscle*.

## Conflicts of Interest

The authors declare no conflicts of interest.

## Supporting information


**Data S1:** Supporting information.

**Table S1:** Antibodies used for immunohistochemistry.
**Table S2:** Presence of abnormal and pathological findings in the vastus lateralis muscles of patients with post‐COVID condition (PCC, *n* = 27). The prevalence of the abnormalities was mostly low, but there was a large variability between patients.
**Table S3:** Muscle fibre characteristics for the post‐COVID condition (PCC) patients (*n* = 27) and age‐ and sex‐matched healthy controls (*n* = 27). Data are presented as means and (SD). Independent Student *t* test or Mann–Whitney *U* was used to assess the differences between the groups. Significance set to *p* < 0.05.
Fig. S1 Mean intensity of LC3B (A) and p62 (B) in myofibers from skeletal muscle of post‐COVID patients (PCC; *n* = 27) and age+/sex‐matched healthy controls (*n* = 27). Representative images of LC3B straining in a patient with PCC (C) and control (D), and p62 staining in a patient with PCC (E) and control (F). Scale bar 100 μm. In (A‐B), mean intensity was measured within each myofiber and averaged for each subject. Data are presented as median and interquartile range (IQR). Mann–Whitney test was used to assess the differences between the groups. Significance set to *p* < 0.05.
**Table S4:** Individual lipid species in skeletal muscle tissue of healthy controls (*n* = 28) and patients with post–COVID‐19 condition (PCC; *n* = 27) determined by targeted lipidomics.
