# Supplementary material for: Muscle Abnormalities in Nonhospitalised Patients With Post–COVID‐19 Condition
Source: J Cachexia Sarcopenia Muscle. 2025 Oct 1;16(5):e70085. doi: 10.1002/jcsm.70085 (PMC12485287; doi:10.1002/jcsm.70085)
Supplement: Supplementary file 2 — Table S1: Antibodies used for immunohistochemistry. Table S2: Presence of abnormal and pathological findings in the vastus lateralis muscles of patients with post‐COVID condition (PCC, n = 27). The prevalence of the abnormalities was mostly low, but there was a large variability between patients. Table S3: Muscle fibre characteristics for the post‐COVID condition (PCC) patients (n = 27) and age‐ and sex‐matched healthy controls (n = 27). Data are presented as means and (SD). Independent Student t test or Mann–Whitney U was used to assess the differences between the groups. Significance set to p < 0.05. Fig. S1 Mean intensity of LC3B (A) and p62 (B) in myofibers from skeletal muscle of post‐COVID patients (PCC; n = 27) and age+/sex‐matched healthy controls (n = 27). Representative images of LC3B straining in a patient with PCC (C) and control (D), and p62 staining in a patient with PCC (E) and control (F). Scale bar 100 μm. In (A‐B), mean intensity was measured within each myofiber and averaged for each subject. Data are presented as median and interquartile range (IQR). Mann–Whitney test was used to assess the differences between the groups. Significance set to p < 0.05. Table S4: Individual lipid species in skeletal muscle tissue of healthy controls (n = 28) and patients with post–COVID‐19 condition (PCC; n = 27) determined by targeted lipidomics. [file JCSM-16-e70085-s001.docx]

**Supplementary Material**

**Table S1:** Antibodies used for immunohistochemistry.

| Antibody ID | Specificity | Gene* | Dilution |  | Source identification |
| --- | --- | --- | --- | --- | --- |
| BA-F8 | MyHC-1 | *MYH7* | 1:100 | mAb-mouse | DSHB Cat# BA-F8, RRID:AB_10 572253 |
| NCL-MHCn | MyHC-neo | *MYH8* | 1:200 | mAb-mouse | Leica Biosystems Cat# NCL-MHCn, RRID:AB_563900 |
| 4C7 | Laminin α5 chain | *LAMA5* | 1:200 | mAb-mouse | Agilent (DAKO) Cat# M0638, RRID:AB_2249754 |
| 11575 | Laminin | *LAMA* | 1:1000 | pAb-rabbit | Abcam Cat# ab11575, RRID:AB 298179 |
| Z0097 | Laminin | *LAMA* | 1:1000 | pAb-rabbit | Agilent (DAKO) Cat#104, RRID: AB_2313665 |
| A0245 | Fibronectin | *FN1* | 1:500 | pAb-rabbit | Agilent (DAKO), Sweden, Cat# A024502, RRID:AB 578510 |
| M0760 | Desmin | *DES* | 1:100 | mAb-mouse | Agilent (DAKO), Cat# M0760, RRID:AB_2335684 |
| CD68 | Macrophage antigen | *CD68* | 1:200 | mAb-mouse | Thermo Fisher Scientific Cat# 14-0688-82, RRID:AB 11151139 |
| LC3B | LC3B | *MAP1LC3B* | 1:100 | pAb-rabbit | Cell Signaling Technology Cat#2775S |
| p62 clone 2C11 | p62 | *SQSTM1* | 1:100 | mAb-mouse | Sigma-Aldrich, Cat#WH0008878M1 |
| *Secondary Antibodies* | | | | | |
| Alexa Fluor™ 488 goat anti-mouse IgG2b | mAb BA-F8 |  | 1:1000 |  | Jackson ImmunoResearch Europe, Ltd, Cambridgeshire, Ely, UK |
| Alexa Fluor™ 488 goat anti-rabbit IgG | Abs A0245, M0760, NCL-MHCn |  | 1:500 |  | Jackson ImmunoResearch Europe, Ltd, Cambridgeshire, Ely, UK |
| Alexa Fluor™ 555 goat anti-mouse IgG2a | 4C7 |  | 1:500 |  | Jackson ImmunoResearch Europe, Ltd, Cambridgeshire, Ely, UK |
| Alexa Fluor™ 568 goat anti-mouse IgG | A-11004 |  | 1:1000 |  | Jackson ImmunoResearch Europe, Ltd, Cambridgeshire, Ely, UK |
| Alexa Fluor™ 647 goat anti-rabbit IgG | Ab 11575 |  | 1:500 |  | Jackson ImmunoResearch Europe, Ltd, Cambridgeshire, Ely, UK |
| Alexa Fluor™ 647 goat anti-mouse IgG | mAb CD68 |  | 1:500 |  | Invitrogen, Carlsbad, CA, USA, D21490 |
| Phalloidin |  |  | 1:400 |  | Jackson ImmunoResearch Europe, Ltd, Cambridgeshire, Ely, UK, A22287 |
| DAPI FluoroPure™ | Nuclear stain |  | 1:1000 |  | Invitrogen, Carlsbad, CA, USA, D21490 |

Mabs BA-F8 are sourced from the Developmental Studies Hybridoma Bank (DSHB), developed under the auspices of the NICHD and maintained by the University of Iowa, Department of Biological Sciences, Iowa City, IA, USA. *Official gene nomenclature is according to OMIM. (<http://www.ncbi.nlm.nih.gov/omim/>)

**Table S2:** Presence of abnormal and pathological findings in the vastus lateralis muscles of patients with post-COVID condition (PCC, N=27). The prevalence of the abnormalities was mostly low, but there was a large variability between patients.

| Case | Atrophic angulated  fibers | Small round fibers^A^ | Small group fiber atrophy | Nuclear clumps^B^ | Fiber hyper-  trophy | Fibrosis | Fat infiltration | Inflammatory cells ^C^ | Fiber  necrosis^D^ | Fetal MyHC^E^ |
| --- | --- | --- | --- | --- | --- | --- | --- | --- | --- | --- |
| 1 |  |  |  | X |  | X |  |  |  |  |
| 2 | X | X | X | X | X | X | X | X |  | X |
| 3 | X |  | X | X | X | X | X | X | X | X |
| 4 | X |  |  |  |  | X | X | X | X |  |
| 5 |  |  |  |  |  |  |  |  |  | X |
| 6 | X | X |  | X | X | X | X |  | X | X |
| 7 | X | X |  | X | X | X | X | X | X | X |
| 8 | X | X |  |  | X | X |  |  | X |  |
| 9 | X |  |  |  |  | X |  |  | X | X |
| 10 | X |  |  | X | X | X | X | X | X | X |
| 11 | X | X |  |  |  | X | X |  | X | X |
| 12 |  |  |  | X |  | X | X |  | X |  |
| 13 | X | X |  | X | X | X |  |  |  |  |
| 14 | X | X |  | X |  | X | X |  | X | X |
| 15 | X | X |  | X |  | X |  |  |  | X |
| 16 | X |  | X | X |  |  |  |  | X | X |
| 17 | X | X |  | X | X | X | X |  | X |  |
| 18 |  |  |  | X |  |  |  |  |  |  |
| 19 | X | X | X | X | X | X | X | X | X | X |
| 20 |  |  |  |  |  | X | X |  |  | X |
| 21 |  |  |  | X |  | X |  |  |  | X |
| 22 | X |  | X | X | X | X |  |  | X | X |
| 23 | X | X | X |  | X | X | X |  | X | X |
| 24 | X | X |  | X |  |  |  |  |  | X |
| 25 |  |  |  |  |  |  |  |  |  | X |
| 26 | X | X | X |  | X | X | X |  | X | X |
| 27 | X | X | X | X | X | X | X | X | X |  |

^A^ CSA <250 μm; ^B^ nuclear clumps indicate severe denervation atrophy; ^C^ accumulation of inflammatory cells in the extracellular matrix; ^D^ Necrotic fibers invaded by phagocytes; ^E^ Muscle fibers co-expressing developmental fetal MyHC

**Table S3:** Muscle fiber characteristics for the post-COVID condition (PCC) patients (N=27) and age- and sex-matched healthy controls (N=27). Data are presented as means and (SD). Independent student t-test or Mann-Whitney U were used to assess the differences between the groups. Significance set to P<0.05.

|  | PCC patients | Controls | P value |
| --- | --- | --- | --- |
| Fiber type proportion, mean (SD), % | | | |
| Type 1 | 49.4 (13.5) | 51.3 (12.5) | 0.594 |
| Type 2 | 50.6 (13.5) | 48.7 (12.5) | 0.594 |
| Muscle fibre CSA, mean (SD), μm^2^ | | | |
| Type 1 | 4553 (1422) | 4932 (1380) | 0.325 |
| Type 2 | 3533 (1249) | 4275 (1646) | 0.068 |
| Average | 4031 (1365) | 4982 (1463) | 0.018 |
| Coefficient variation CSA, mean % (SD) | | | |
| Type 1 | 49.9 (9.0) | 49.4 (11.0) | 0.860 |
| Type 2 | 56.4 (8.7) | 54.8 (10.5) | 0.549 |
| Average | 55.4 (9.0) | 54.0 (10.1) | 0.572 |
| Capillary supply per area, mean (SD), number of supplying capillaries x mm^−2^ | | | |
| Type 1 | 895 (229) | 982 (275) | 0.212 |
| Type 2 | 1020 (385) | 1066 (385) | 0.659 |
| Average | 925 (241) | 997 (262) | 0.300 |
| Capillary-to-fiber ratio, mean (SD), ratio | | | |
| Type 1 | 2.2 (0.7) | 2.6 (0.9) | 0.044 |
| Type 2 | 1.8 (0.6) | 2.2 (0.8) | 0.022 |
| Average | 2.0 (0.6) | 2.4 (0.8) | 0.024 |
| Capillary contacts, mean (SD), number | | | |
| Type 1 | 7.2 (1.7) | 7.9 (1.4) | 0.077 |
| Type 2 | 6.0 (1.5) | 6.8 (1.6) | 0.080 |
| Average | 6.6 (1.7) | 7.4 (1.4) | 0.049 |
| CFPE, mean (SD), capillaries x 1000 μm^−1^ | | | |
| Type 1 | 8.0 (1.8) | 9.3 (2.8) | 0.052 |
| Type 2 | 7.6 (2.0) | 8.7 (2.5) | 0.073 |
| Average | 7.8 (1.9) | 9.0 (2.7) | 0.050 |
| Myonuclei per fiber, mean (SD), number | 3.4 (1.1) | 4.1 (1.0) | 0.012 |
| Myonuclei per fiber area, mean (SD), number x μm^−1^ | 0.0009 (0.00019) | 0.0008 (0.00013) | 0.902 |
| CD68+ per fiber, mean (SD), number | 0.28 (0.15) | 0.22 (1.0) | 0.115 |
| CD68+ per fiber area, mean (SD), number x μm^−1^ | 0.0001 (0.00007) | 0.0004 (0.0002) | 0.004 |
| Myofiber LC3, mean (SD), AU | 0.0032 (0.0007) | 0.0030 (0.0006) | 0.232 |
| Myofiber p62, mean (SD), AU | 0.0079 (0.0023) | 0.0079 (0.0016) | 0.814 |

CSA; cross-sectional area, CFPE; capillary-to-fiber perimeter exchange; AU: Arbitrary units

**Fig. S1:** Mean intensity of LC3B (A) and p62 (B) in myofibers from skeletal muscle of post-COVID patients (PCC; n=27) and age-/sex-matched healthy controls (n=27). Representative images of LC3B straining in a patient with PCC (C) and control (D), and p62 staining in a patient with PCC (E) and control (F). Scale bar 100μm. In (A-B), mean intensity was measured within each myofiber and averaged for each subject. Data are presented as median and interquartile range (IQR). Mann-Whitney test was used to assess the differences between the groups. Significance set to P<0.05.

**Table S4:** Individual lipid species in skeletal muscle tissue of healthy controls (N=28) and patients with post-COVID-19 Condition (PCC; N=27) determined by targeted lipidomics.

| LIPIDS (µg / mg tissue) | Controls | PCC | *p-val* | *q-val* |
| --- | --- | --- | --- | --- |
| *PHOSPHATIDYLCHOLINES* |  |  |  |  |
| PC28:0(14:0_14:0) | 0.300 ± 0.167 | 0.252 ± 0.121 | 0.228 | 0.466 |
| PC28:0(16:0_12:0) | 0.094 ± 0.070 | 0.099 ± 0.065 | 0.781 | 0.875 |
| PC30:0(16:0_14:0) | 0.466 ± 0.209 | 0.456 ± 0.170 | 0.845 | 0.924 |
| PC32:0(14:0_18:0) | 0.028 ± 0.025 | 0.018 ± 0.015 | 0.080 | 0.351 |
| PC32:0(16:0_16:0) | 1.233 ± 0.518 | 1.181 ± 0.412 | 0.687 | 0.823 |
| PC33:0(16:0_17:0) | 0.015 ± 0.022 | 0.016 ± 0.018 | 0.890 | 0.951 |
| PC34:0(16:0_18:0) | 0.116 ± 0.053 | 0.127 ± 0.050 | 0.421 | 0.601 |
| PC36:0(18:0_18:0) | 0.020 ± 0.017 | 0.029 ± 0.040 | 0.243 | 0.476 |
| PC30:1(12:0_18:1) | 0.083 ± 0.052 | 0.066 ± 0.046 | 0.203 | 0.433 |
| PC30:1(14:0_16:1) | 0.259 ± 0.144 | 0.257 ± 0.133 | 0.955 | 0.974 |
| PC32:1(14:0_18:1) | 0.798 ± 0.373 | 0.772 ± 0.224 | 0.758 | 0.869 |
| PC32:1(16:0_16:1) | 0.987 ± 0.460 | 1.087 ± 0.525 | 0.454 | 0.622 |
| PC33:1(15:0_18:1) | 0.079 ± 0.052 | 0.088 ± 0.045 | 0.506 | 0.669 |
| PC34:1(16:0_18:1) | 9.760 ±2.520 | 10.312 ± 1.941 | 0.368 | 0.561 |
| PC34:1(18:0_16:1) | 0.063 ± 0.040 | 0.063 ± 0.040 | 0.997 | >0.999 |
| PC35:1(17:0_18:1) | 0.095 ± 0.055 | 0.085 ± 0.047 | 0.474 | 0.638 |
| PC36:1(16:0_20:1) | 0.111 ± 0.058 | 0.114 ± 0.058 | 0.848 | 0.924 |
| PC36:1(18:0_18:1) | 3.265 ± 1.149 | 3.599 ± 0.924 | 0.241 | 0.476 |
| PC38:1(18:0_20:1) | 0.024 ± 0.022 | 0.018 ± 0.018 | 0.340 | 0.541 |
| PC38:1(20:0_18:1) | 0.005 ± 0.009 | 0.009 ± 0.013 | 0.184 | 0.433 |
| PC32:2(14:0_18:2) | 6.064 ± 1.821 | 5.185 ± 1.668 | 0.068 | 0.314 |
| PC32:2(16:1_16:1) | 0.162 ± 0.125 | 0.189 ± 0.117 | 0.425 | 0.603 |
| PC33:2(15:0_18:2) | 0.812 ± 0.228 | 0.628 ± 0.211 | **0.003** | 0.062 |
| PC34:2(16:0_18:2) | 23.136 ± 5.623 | 23.162 ± 4.516 | 0.985 | 0.995 |
| PC34:2(16:1_18:1) | 0.321 ± 0.167 | 0.361 ± 0.152 | 0.367 | 0.561 |
| PC35:2(17:0_18:2) | 0.348 ± 0.133 | 0.302 ± 0.080 | 0.133 | 0.415 |
| PC36:2(16:0_20:2) | 0.046 ± 0.028 | 0.036 ± 0.023 | 0.148 | 0.415 |
| PC36:2(18:0_18:2) | 9.031 ± 2.405 | 9.110 ± 2.002 | 0.896 | 0.953 |
| PC36:2(18:1_18:1) | 2.312 ± 0.841 | 2.681 ± 0.577 | 0.064 | 0.314 |
| PC38:2(18:0_20:2) | 0.025 ± 0.016 | 0.026 ± 0.021 | 0.791 | 0.879 |
| PC38:2(18:1_20:1) | 0.014 ± 0.017 | 0.011 ± 0.009 | 0.339 | 0.541 |
| PC38:2(20:0_18:2) | 0.132 ± 0.089 | 0.137 ± 0.082 | 0.821 | 0.901 |
| PC32:3(14:0_18:3) | 0.298 ± 0.157 | 0.249 ± 0.139 | 0.230 | 0.466 |
| PC34:3(14:0_20:3) | 0.063 ± 0.029 | 0.048 ± 0.033 | 0.082 | 0.352 |
| PC34:3(16:0_18:3) | 3.324 ± 0.914 | 3.061 ± 0.847 | 0.274 | 0.491 |
| PC34:3(16:1_18:2) | 2.649 ± 0.813 | 2.668 ± 0.875 | 0.931 | 0.965 |
| PC35:3(17:1_18:2) | 0.048 ± 0.027 | 0.043 ± 0.023 | 0.473 | 0.638 |
| PC36:3(16:0_20:3) | 1.218 ± 0.471 | 1.073 ± 0.301 | 0.182 | 0.433 |
| PC36:3(18:0_18:3) | 0.208 ± 0.104 | 0.164 ± 0.062 | 0.066 | 0.314 |
| PC36:3(18:1_18:2) | 3.090 ± 1.029 | 3.329 ± 0.804 | 0.343 | 0.543 |
| PC38:3(18:0_20:3) | 0.396 ± 0.124 | 0.400 ± 0.146 | 0.917 | 0.965 |
| PC34:4(14:0_20:4) | 0.514 ± 0.228 | 0.476 ± 0.203 | 0.519 | 0.679 |
| PC34:4(16:1_18:3) | 0.164 ± 0.116 | 0.153 ± 0.106 | 0.721 | 0.840 |
| PC35:4(15:0_20:4) | 0.014 ± 0.013 | 0.016 ± 0.018 | 0.669 | 0.807 |
| PC36:4(16:0_20:4) | 7.443 ± 2.507 | 6.810 ± 2.415 | 0.345 | 0.544 |
| PC36:4(18:1_18:3) | 0.567 ± 0.233 | 0.541 ± 0.189 | 0.657 | 0.798 |
| PC36:4(18:2_18:2) | 2.214 ± 1.000 | 2.067 ± 0.892 | 0.568 | 0.724 |
| PC38:4(16:0_22:4) | 0.897 ± 0.634 | 0.612 ± 0.329 | **0.042** | 0.285 |
| PC38:4(18:0_20:4) | 3.112 ± 1.206 | 2.941 ± 1.086 | 0.584 | 0.740 |
| PC38:4(18:1_20:3) | 0.240 ± 0.107 | 0.213 ± 0.070 | 0.275 | 0.491 |
| PC40:4(18:0_22:4) | 0.175 ± 0.077 | 0.159 ± 0.080 | 0.439 | 0.617 |
| PC40:4(20:0_20:4) | 0.161 ± 0.103 | 0.148 ± 0.098 | 0.639 | 0.781 |
| PC34:5(14:0_20:5) | 0.102 ± 0.101 | 0.071 ± 0.050 | 0.164 | 0.415 |
| PC36:5(16:0_20:5) | 2.200 ± 1.306 | 2.054 ± 0.901 | 0.633 | 0.779 |
| PC36:5(18:2_18:3) | 0.167 ± 0.091 | 0.148 ± 0.115 | 0.511 | 0.670 |
| PC38:5(16:0_22:5) | 1.110 ± 0.405 | 0.971 ± 0.313 | 0.161 | 0.415 |
| PC38:5(18:0_20:5) | 0.210 ± 0.132 | 0.229 ± 0.105 | 0.554 | 0.710 |
| PC38:5(18:1_20:4) | 1.817 ± 0.622 | 1.765 ± 0.726 | 0.776 | 0.875 |
| PC38:5(18:2_20:3) | 0.029 ± 0.024 | 0.029 ± 0.020 | 0.956 | 0.974 |
| PC40:5(18:0_22:5) | 0.308 ± 0.127 | 0.294 ± 0.104 | 0.669 | 0.807 |
| PC40:5(18:1_22:4) | 0.138 ± 0.104 | 0.092 ± 0.067 | 0.060 | 0.314 |
| PC36:6(14:0_22:6) | 0.063 ± 0.051 | 0.049 ± 0.036 | 0.236 | 0.472 |
| PC36:6(16:1_20:5) | 0.060 ± 0.062 | 0.058 ± 0.041 | 0.891 | 0.951 |
| PC38:6(16:0_22:6) | 2.466 ± 1.116 | 2.005 ± 0.897 | 0.098 | 0.383 |
| PC38:6(18:1_20:5) | 0.480 ± 0.264 | 0.498 ± 0.256 | 0.803 | 0.886 |
| PC38:6(18:2_20:4) | 0.261 ± 0.099 | 0.255 ± 0.123 | 0.852 | 0.925 |
| PC40:6(18:0_22:6) | 0.488 ± 0.342 | 0.430 ± 0.261 | 0.482 | 0.646 |
| PC40:6(18:1_22:5) | 0.161 ± 0.062 | 0.158 ± 0.087 | 0.907 | 0.962 |
| PC42:6(20:0_22:6) | 0.042 ± 0.025 | 0.040 ± 0.028 | 0.711 | 0.835 |
| PC38:7(16:1_22:6) | 0.021 ± 0.024 | 0.024 ± 0.028 | 0.621 | 0.775 |
| PC38:7(18:2_20:5) | 0.043 ± 0.029 | 0.044 ± 0.052 | 0.929 | 0.965 |
| PC40:7(18:1_22:6) | 0.576 ± 0.312 | 0.520 ± 0.298 | 0.503 | 0.669 |
| PC40:8(18:2_22:6) | 0.051 ± 0.033 | 0.058 ± 0.044 | 0.555 | 0.710 |
| *PLASMENYL-PHOSPHATIDYLCHOLINES* |  |  |  |  |
| PC(P30:0) (P16:0_14:0) | 0.027 ± 0.022 | 0.027 ± 0.027 | 0.941 | 0.968 |
| PC(P32:0) (P16:0_16:0) | 0.079 ± 0.06 | 0.072 ± 0.082 | 0.713 | 0.835 |
| PC(P32:0) (P16:0_16:1) | 0.072 ± 0.054 | 0.046 ± 0.037 | **0.045** | 0.286 |
| PC(P34:0) (P16:0_18:0) | 5.505 ± 2.521 | 4.306 ± 1.872 | 0.051 | 0.308 |
| PC(P34:0) (P18:0_16:0) | 0.232 ± 0.100 | 0.301 ± 0.187 | 0.090 | 0.358 |
| PC(P34:1) (P16:0_18:1) | 1.755 ± 0.910 | 1.078 ± 0.498 | **0.001** | **0.035** |
| PC(P34:1) (P18:0_16:1) | 0.206 ± 0.152 | 0.157 ± 0.141 | 0.219 | 0.458 |
| PC(P34:1) (P18:1_16:0) | 0.850 ± 0.507 | 0.566 ± 0.358 | **0.020** | 0.179 |
| PC(P36:1) (P18:0_18:1) | 0.599 ± 0.264 | 0.459 ± 0.246 | **0.046** | 0.286 |
| PC(P36:1) (P18:1_18:0) | 0.381 ± 0.179 | 0.277 ± 0.155 | **0.026** | 0.211 |
| PC(P34:2) (P16:0_18:2) | 3.521 ± 5.554 | 1.433 ± 1.904 | 0.070 | 0.314 |
| PC(P36:2) (P18:0_18:2) | 21.054 ± 8.863 | 14.942 ± 7.46 | **0.008** | 0.109 |
| PC(P36:2) (P18:1_18:1) | 2.051 ± 1.108 | 1.582 ± 1.067 | 0.116 | 0.415 |
| PC(P38:2) (P20:0_18:2) | 0.019 ± 0.017 | 0.015 ± 0.014 | 0.395 | 0.577 |
| PC(P34:3) (P16:0_18:3) | 0.045 ± 0.042 | 0.031 ± 0.036 | 0.192 | 0.433 |
| PC(P36:3) (P16:0_20:3) | 0.151 ± 0.184 | 0.071 ± 0.066 | **0.037** | 0.275 |
| PC(P36:3) (P18:0_18:3) | 0.103 ± 0.109 | 0.062 ± 0.042 | 0.070 | 0.314 |
| PC(P38:3) (P18:0_20:3) | 1.662 ± 0.635 | 1.096 ± 0.473 | **0.001** | **0.019** |
| PC(P36:4) (P16:0_20:4) | 3.895 ± 1.832 | 2.325 ± 0.961 | **0.000** | **0.012** |
| PC(P38:4) (P16:0_22:4) | 0.051 ± 0.028 | 0.041 ± 0.036 | 0.254 | 0.487 |
| PC(P38:4) (P18:0_20:4) | 65.95 ± 24.869 | 43.522 ± 15.547 | **0.000** | **0.012** |
| PC(P40:4) (P18:0_22:4) | 0.132 ± 0.065 | 0.116 ± 0.076 | 0.397 | 0.577 |
| PC(P40:4) (P20:0_20:4) | 0.073 ± 0.049 | 0.048 ± 0.028 | **0.028** | 0.218 |
| PC(P36:5) (P16:0_20:5) | 0.232 ± 0.211 | 0.182 ± 0.098 | 0.261 | 0.489 |
| PC(P38:5) (P18:0_20:5) | 2.040 ± 1.480 | 1.589 ± 0.746 | 0.162 | 0.415 |
| PC(P38:6) (P16:0_22:6) | 0.309 ± 0.252 | 0.251 ± 0.162 | 0.318 | 0.527 |
| PC(P40:6) (P18:0_22:6) | 2.520 ± 1.416 | 2.030 ± 0.948 | 0.139 | 0.415 |
| *PHOSPHATIDYLETHANOLAMINES* |  |  |  |  |
| PE32:0(16:0_16:0) | 0.006 ± 0.003 | 0.005 ± 0.003 | 0.279 | 0.494 |
| PE34:0(18:0_16:0) | 0.008 ± 0.005 | 0.005 ± 0.003 | **0.027** | 0.213 |
| PE35:0(18:0_17:0) | 0.001 ± 0.002 | 0.001 ± 0.001 | 0.350 | 0.547 |
| PE36:0(18:0_18:0) | 0.018 ± 0.007 | 0.014 ± 0.005 | **0.023** | 0.195 |
| PE30:1(16:0_14:0) | 0.009 ± 0.008 | 0.004 ± 0.005 | **0.018** | 0.161 |
| PE32:1(14:0_18:1) | 0.008 ± 0.009 | 0.005 ± 0.006 | 0.155 | 0.415 |
| PE32:1(16:0_16:1) | 0.003 ± 0.002 | 0.004 ± 0.003 | 0.504 | 0.669 |
| PE34:1(16:0_18:1) | 0.089 ± 0.031 | 0.075 ± 0.026 | 0.067 | 0.314 |
| PE34:1(18:0_16:1) | 0.003 ± 0.002 | 0.003 ± 0.003 | 0.324 | 0.531 |
| PE36:1(16:0_20:1) | 0.004 ± 0.002 | 0.004 ± 0.002 | 0.724 | 0.840 |
| PE36:1(18:0_18:1) | 0.474 ± 0.156 | 0.393 ± 0.131 | **0.044** | 0.285 |
| PE40:1(22:0_18:1) | 0.043 ± 0.024 | 0.056 ± 0.034 | 0.106 | 0.398 |
| PE32:2(14:0_18:2) | 0.023 ± 0.015 | 0.017 ± 0.008 | 0.069 | 0.314 |
| PE34:2(16:0_18:2) | 0.270 ± 0.104 | 0.200 ± 0.046 | **0.002** | 0.052 |
| PE34:2(18:1_16:1) | 0.004 ± 0.003 | 0.004 ± 0.004 | 0.781 | 0.875 |
| PE36:2(18:0_18:2) | 3.290 ± 1.261 | 2.608 ± 0.709 | **0.017** | 0.159 |
| PE36:2(18:1_18:1) | 0.163 ± 0.070 | 0.158 ± 0.058 | 0.759 | 0.869 |
| PE37:2(17:0_20:2) | 0.003 ± 0.003 | 0.003 ± 0.002 | 0.608 | 0.764 |
| PE38:2(18:0_20:2) | 0.011 ± 0.005 | 0.009 ± 0.005 | 0.089 | 0.358 |
| PE38:2(18:1_20:1) | 0.012 ± 0.009 | 0.009 ± 0.005 | 0.143 | 0.415 |
| PE34:3(16:0_18:3) | 0.040 ± 0.019 | 0.025 ± 0.016 | **0.003** | 0.062 |
| PE34:3(18:2_16:1) | 0.026 ± 0.019 | 0.022 ± 0.012 | 0.284 | 0.501 |
| PE36:3(16:0_20:3) | 0.030 ± 0.014 | 0.019 ± 0.016 | **0.005** | 0.074 |
| PE36:3(18:0_18:3) | 0.109 ± 0.056 | 0.067 ± 0.039 | **0.003** | 0.057 |
| PE36:3(18:1_18:2) | 1.300 ± 0.854 | 0.965 ± 0.378 | 0.067 | 0.314 |
| PE38:3(18:0_20:3) | 0.166 ± 0.079 | 0.129 ± 0.052 | **0.042** | 0.285 |
| PE38:3(18:1_20:2) | 0.003 ± 0.003 | 0.003 ± 0.003 | 0.723 | 0.840 |
| PE38:3(18:2_20:1) | 0.003 ± 0.002 | 0.003 ± 0.002 | 0.348 | 0.546 |
| PE34:4(14:0_20:4) | 0.021 ± 0.012 | 0.014 ± 0.006 | **0.007** | 0.101 |
| PE36:4(16:0_20:4) | 0.250 ± 0.074 | 0.194 ± 0.053 | **0.002** | 0.052 |
| PE36:4(18:1_18:3) | 0.119 ± 0.076 | 0.079 ± 0.055 | **0.031** | 0.235 |
| PE36:4(18:2_18:2) | 0.518 ± 0.289 | 0.344 ± 0.227 | **0.016** | 0.159 |
| PE37:4(17:0_20:4) | 0.049 ± 0.024 | 0.027 ± 0.015 | **0.000** | **0.012** |
| PE38:4(16:0_22:4) | 0.017 ± 0.009 | 0.009 ± 0.005 | **0.000** | **0.012** |
| PE38:4(18:0_20:4) | 8.945 ± 3.235 | 6.790 ± 1.892 | **0.004** | 0.074 |
| PE38:4(18:1_20:3) | 0.056 ± 0.026 | 0.038 ± 0.026 | **0.017** | 0.159 |
| PE40:4(18:0_22:4) | 0.153 ± 0.051 | 0.117 ± 0.046 | **0.009** | 0.112 |
| PE34:5(14:0_20:5) | 0.002 ± 0.003 | 0.001 ± 0.001 | 0.054 | 0.314 |
| PE36:5(16:0_20:5) | 0.055 ± 0.030 | 0.038 ± 0.015 | **0.009** | 0.112 |
| PE38:5(16:0_22:5) | 0.010 ± 0.006 | 0.008 ± 0.004 | 0.060 | 0.314 |
| PE38:5(18:0_20:5) | 0.418 ± 0.197 | 0.301 ± 0.142 | **0.016** | 0.159 |
| PE38:5(18:1_20:4) | 0.482 ± 0.213 | 0.347 ± 0.107 | **0.005** | 0.074 |
| PE38:5(18:2_20:3) | 0.002 ± 0.002 | 0.002 ± 0.001 | 0.086 | 0.358 |
| PE40:5(18:0_22:5) | 0.031 ± 0.026 | 0.037 ± 0.027 | 0.466 | 0.633 |
| PE40:5(18:1_22:4) | 0.009 ± 0.005 | 0.007 ± 0.004 | 0.134 | 0.415 |
| PE36:6(14:0_22:6) | 0.002 ± 0.002 | 0.001 ± 0.001 | 0.083 | 0.352 |
| PE38:6(16:0_22:6) | 0.040 ± 0.018 | 0.029 ± 0.014 | **0.023** | 0.195 |
| PE38:6(18:1_20:5) | 0.104 ± 0.056 | 0.070 ± 0.036 | **0.009** | 0.112 |
| PE38:6(18:2_20:4) | 0.097 ± 0.044 | 0.061 ± 0.024 | **0.001** | **0.019** |
| PE40:6(18:0_22:6) | 0.528 ± 0.197 | 0.370 ± 0.138 | **0.001** | **0.035** |
| PE40:6(18:1_22:5) | 0.022 ± 0.012 | 0.017 ± 0.007 | **0.043** | 0.285 |
| PE38:7(18:2_20:5) | 0.007 ± 0.006 | 0.005 ± 0.006 | 0.145 | 0.415 |
| PE40:7(18:1_22:6) | 0.053 ± 0.032 | 0.040 ± 0.018 | 0.069 | 0.314 |
| PE40:7(18:2_22:5) | 0.008 ± 0.005 | 0.005 ± 0.003 | **0.011** | 0.124 |
| *PLASMENYL-PHOSPHATIDYLETHANOLAMINES* |  |  |  |  |
| PE(P34:1) P-16:0_18:1 | 0.915 ± 0.414 | 0.925 ± 0.395 | 0.926 | 0.965 |
| PE(P36:1) P-18:0_18:1 | 0.775 ± 0.302 | 0.953 ± 0.387 | **0.063** | 0.314 |
| PE(P34:2) P-16:0_18:2 | 5.186 ± 2.417 | 4.643 ± 2.352 | 0.403 | 0.584 |
| PE(P36:2) P-18:0_18:2 | 6.594 ± 2.704 | 6.834 ± 3.513 | 0.778 | 0.875 |
| PE(P36:2) P-18:1_18:1 | 0.235 ± 0.126 | 0.290 ± 0.137 | 0.131 | 0.415 |
| PE(P38:2) P-20:0_18:2 | 0.074 ± 0.043 | 0.071 ± 0.041 | 0.765 | 0.874 |
| PE(P36:3) P-18:1_18:2 | 3.089 ± 1.285 | 3.345 ± 1.712 | 0.533 | 0.694 |
| PE(P38:3) P-18:0_20:3 | 1.744 ± 0.640 | 1.792 ± 0.750 | 0.796 | 0.881 |
| PE(P36:4) P-16:0_20:4 | 19.152 ± 7.197 | 14.488 ± 5.615 | **0.010** | 0.120 |
| PE(P38:4) P-16:0_22:4 | 1.209 ± 0.465 | 1.175 ± 0.583 | 0.816 | 0.898 |
| PE(P38:4) P-18:0_20:4 | 13.937 ± 5.349 | 13.825 ± 6.486 | 0.944 | 0.968 |
| PE(P40:4) P-18:0_22:4 | 1.420 ± 0.498 | 1.373 ± 0.714 | 0.780 | 0.875 |
| PE(P40:4) P-20:0_20:4 | 0.272 ± 0.139 | 0.236 ± 0.130 | 0.320 | 0.528 |
| PE(P40:4) P-20:1_20:3 | 0.012 ± 0.008 | 0.007 ± 0.004 | **0.017** | 0.159 |
| PE(P36:5) P16:0_20:5 | 0.423 ± 0.308 | 0.371 ± 0.198 | 0.459 | 0.627 |
| PE(P38:5) P-16:0_22:5 | 1.833 ± 0.666 | 1.412 ± 0.579 | **0.016** | 0.159 |
| PE(P38:5) P-18:0_20:5 | 0.352 ± 0.208 | 0.344 ± 0.165 | 0.872 | 0.942 |
| PE(P38:5) P-18:1_20:4 | 4.432 ± 1.561 | 3.883 ± 1.354 | 0.170 | 0.421 |
| PE(P40:5) P-18:0_22:5 | 0.744 ± 0.309 | 0.751 ± 0.370 | 0.941 | 0.968 |
| PE(P40:5) P-20:1_20:4 | 0.070 ± 0.042 | 0.061 ± 0.028 | 0.331 | 0.537 |
| PE(P38:6) P-16:0_22:6 | 0.688 ± 0.418 | 0.599 ± 0.304 | 0.374 | 0.561 |
| PE(P40:6) P-18:0_22:6 | 0.455 ± 0.255 | 0.490 ± 0.280 | 0.632 | 0.779 |
| PE(P40:7) P-18:1_22:6 | 0.333 ± 0.195 | 0.327 ± 0.172 | 0.910 | 0.962 |
| *PHOSPHATIDYLINOSITOLS* |  |  |  |  |
| PI34:1(16:0_18:1) | 0.002 ± 0.002 | 0.002 ± 0.002 | 0.710 | 0.835 |
| PI36:4(16:0_20:4) | 0.002 ± 0.004 | 0.001 ± 0.002 | 0.379 | 0.561 |
| PI38:4(18:0_20:4) | 0.077 ± 0.041 | 0.069 ± 0.028 | 0.442 | 0.618 |
| PI38:5(18:1_20:4) | 0.008 ± 0.005 | 0.007 ± 0.007 | 0.737 | 0.852 |
| *PHOSPHATIDYLSERINES* |  |  |  |  |
| PS34:1(16:0_18:1) | 0.008 ± 0.008 | 0.003 ± 0.004 | **0.013** | 0.146 |
| PS34:2(16:0_18:2) | 0.002 ± 0.002 | 0.002 ± 0.001 | 0.231 | 0.466 |
| PS36:2(18:1_18:1) | 0.014 ± 0.009 | 0.010 ± 0.007 | 0.172 | 0.421 |
| PS36:2(20:1_16:1) | 0.027 ± 0.016 | 0.018 ± 0.016 | **0.043** | 0.285 |
| PS36:2(20:2_16:0) | 0.082 ± 0.038 | 0.062 ± 0.036 | **0.051** | 0.308 |
| PS36:3(18:1_18:2) | 0.037 ± 0.041 | 0.026 ± 0.021 | 0.200 | 0.433 |
| PS38:3(18:0_20:3) | 0.016 ± 0.011 | 0.012 ± 0.008 | 0.114 | 0.412 |
| PS38:4(18:0_20:4) | 0.032 ± 0.026 | 0.029 ± 0.021 | 0.614 | 0.769 |
| PS38:4(16:0_22:4) | 0.202 ± 0.121 | 0.106 ± 0.063 | **0.001** | **0.019** |
| PS38:4(18:1_20:3) | 0.009 ± 0.010 | 0.006 ± 0.005 | 0.161 | 0.415 |
| PS40:4(20:0_20:4) | 7.477 ± 2.625 | 4.601 ± 1.940 | **0.000** | **0.009** |
| PS38:5(18:1_20:4) | 0.003 ± 0.004 | 0.002 ± 0.003 | 0.274 | 0.491 |
| PS38:5(18:0_20:5) | 0.006 ± 0.006 | 0.005 ± 0.004 | 0.417 | 0.598 |
| PS40:5(20:0_20:5) | 0.361 ± 0.249 | 0.256 ± 0.158 | 0.070 | 0.314 |
| PS40:5(20:1_20:4) | 1.633 ± 0.756 | 0.914 ± 0.578 | **0.000** | **0.012** |
| PS40:6(18:0_22:6) | 0.044 ± 0.061 | 0.044 ± 0.068 | 0.984 | 0.995 |
| *CERAMIDES* |  |  |  |  |
| Cer34:1(d18:1_c16:0) | 0.315 ± 0.261 | 0.360 ± 0.287 | 0.539 | 0.700 |
| Cer36:1(d18:1_c18:0) | 7.435 ± 3.526 | 8.533 ± 4.121 | 0.293 | 0.504 |
| Cer40:1(d18:1_c22:0) | 1.550 ± 1.053 | 1.111 ± 0.646 | 0.070 | 0.314 |
| Cer41:1(d18:1_c23:0) | 0.596 ± 0.514 | 0.423 ± 0.413 | 0.177 | 0.425 |
| Cer42:1(d18:1_c24:0) | 1.589 ± 0.872 | 1.338 ± 0.876 | 0.292 | 0.504 |
| Cer40:2(d18:2_c22:0) | 0.213 ± 0.173 | 0.189 ± 0.193 | 0.634 | 0.779 |
| Cer42:2(d18:1_c24:1) | 1.977 ± 1.185 | 1.870 ± 0.841 | 0.701 | 0.832 |
| Cer42:3(d18:2_c24:1) | 0.265 ± 0.266 | 0.290 ± 0.214 | 0.700 | 0.832 |
| *SPHINGOMYELINS* |  |  |  |  |
| SM(14:0) | 2.927 ± 3.952 | 2.059 ± 2.514 | 0.337 | 0.541 |
| SM(16:0) | 3.912 ± 4.382 | 2.854 ± 3.521 | 0.329 | 0.535 |
| SM(18:0) | 2.147 ± 2.206 | 1.724 ± 1.838 | 0.445 | 0.620 |
| SM(20:0) | 2.146 ± 2.723 | 1.431 ± 1.434 | 0.231 | 0.466 |
| SM(22:0) | 7.727 ± 8.124 | 5.726 ± 4.783 | 0.273 | 0.491 |
| SM(24:0) | 6.725 ± 6.862 | 4.585 ± 3.857 | 0.162 | 0.415 |
| SM(26:0) | 0.090 ± 0.082 | 0.052 ± 0.042 | **0.038** | 0.276 |
| SM(18:1) | 0.332 ± 0.333 | 0.305 ± 0.289 | 0.749 | 0.863 |
| SM(20:1) | 0.250 ± 0.250 | 0.152 ± 0.157 | 0.089 | 0.358 |
| SM(22:1) | 2.525 ± 2.866 | 1.818 ± 1.681 | 0.271 | 0.491 |
| SM(24:1) | 11.937 ± 11.915 | 9.594 ± 9.954 | 0.433 | 0.611 |
| SM(26:1) | 0.228 ± 0.267 | 0.136 ± 0.147 | 0.123 | 0.415 |
| *TRIGLYCERIDES* |  |  |  |  |
| TAG(42:0) 12:0_14:0_16:0 | 0.112 ± 0.086 | 0.074 ± 0.060 | 0.065 | 0.314 |
| TAG(44:0) 14:0_14:0_16:0 | 0.074 ± 0.036 | 0.070 ± 0.050 | 0.771 | 0.875 |
| TAG(46:0) 14:0_16:0_16:0 | 0.117 ± 0.055 | 0.111 ± 0.066 | 0.704 | 0.833 |
| TAG(48:0) 16:0_16:0_16:0 | 0.230 ± 0.129 | 0.225 ± 0.118 | 0.872 | 0.942 |
| TAG(50:0) 16:0_16:0_18:0 | 0.413 ± 0.240 | 0.408 ± 0.268 | 0.945 | 0.968 |
| TAG(52:0) 16:0_16:0_20:0 | 0.032 ± 0.019 | 0.029 ± 0.022 | 0.542 | 0.700 |
| TAG(52:0) 16:0_18:0_18:0 | 0.695 ± 0.487 | 0.675 ± 0.500 | 0.880 | 0.945 |
| TAG(54:0) 18:0_18:0_18:0 | 0.177 ± 0.213 | 0.130 ± 0.172 | 0.373 | 0.561 |
| TAG(42:1) 12:0_12:0_18:1 | 0.245 ± 0.383 | 0.110 ± 0.128 | 0.087 | 0.358 |
| TAG(44:1) 12:0_14:0_18:1 | 0.250 ± 0.230 | 0.164 ± 0.151 | 0.105 | 0.398 |
| TAG(44:1) 14:0_14:0_16:1 | 0.101 ± 0.093 | 0.072 ± 0.073 | 0.201 | 0.433 |
| TAG(44:1) 14:0_14:1_16:0 | 0.289 ± 0.273 | 0.193 ± 0.192 | 0.140 | 0.415 |
| TAG(46:1) 12:0_16:0_18:1 | 0.216 ± 0.089 | 0.183 ± 0.101 | 0.201 | 0.433 |
| TAG(46:1) 14:0_16:0_16:1 | 0.158 ± 0.078 | 0.133 ± 0.092 | 0.267 | 0.491 |
| TAG(48:1) 14:0_16:0_18:1 | 0.328 ± 0.161 | 0.305 ± 0.193 | 0.634 | 0.779 |
| TAG(48:1) 14:0_16:1_18:0 | 0.051 ± 0.026 | 0.050 ± 0.034 | 0.878 | 0.945 |
| TAG(49:1) 15:0_16:0_18:1 | 0.343 ± 0.327 | 0.201 ± 0.155 | **0.046** | 0.286 |
| TAG(49:1) 15:0_16:1_18:0 | 0.231 ± 0.192 | 0.176 ± 0.151 | 0.247 | 0.477 |
| TAG(50:1) 16:0_16:0_18:1 | 0.498 ± 0.258 | 0.443 ± 0.239 | 0.415 | 0.598 |
| TAG(50:1) 16:1_16:0_18:0 | 0.071 ± 0.028 | 0.062 ± 0.032 | 0.304 | 0.516 |
| TAG(51:1) 15:0_18:1_18:0 | 0.363 ± 0.393 | 0.358 ± 0.374 | 0.960 | 0.975 |
| TAG(51:1) 16:0_17:0_18:1 | 0.702 ± 0.735 | 0.720 ± 0.761 | 0.930 | 0.965 |
| TAG(52:1) 16:0_18:0_18:1 | 1.074 ± 0.757 | 0.904 ± 0.671 | 0.381 | 0.562 |
| TAG(53:1) 17:0_18:0_18:1 | 0.054 ± 0.067 | 0.046 ± 0.041 | 0.632 | 0.779 |
| TAG(54:1) 16:0_18:1_20:0 | 0.192 ± 0.244 | 0.121 ± 0.116 | 0.177 | 0.425 |
| TAG(54:1) 18:0_18:0_18:1 | 0.722 ± 1.076 | 0.308 ± 0.318 | 0.060 | 0.314 |
| TAG(56:1) 16:0_18:1_22:0 | 0.139 ± 0.174 | 0.083 ± 0.068 | 0.123 | 0.415 |
| TAG(56:1) 16:1_18:0_22:0 | 0.180 ± 0.271 | 0.093 ± 0.078 | 0.111 | 0.412 |
| TAG(44:2) 12:0_14:0_18:2 | 0.193 ± 0.301 | 0.079 ± 0.097 | 0.066 | 0.314 |
| TAG(44:2) 12:0_14:1_18:1 | 0.235 ± 0.375 | 0.124 ± 0.184 | 0.172 | 0.421 |
| TAG(44:2) 14:1_14:1_16:0 | 0.228 ± 0.344 | 0.118 ± 0.181 | 0.148 | 0.415 |
| TAG(46:2) 12:0_16:0_18:2 | 0.304 ± 0.297 | 0.198 ± 0.181 | 0.119 | 0.415 |
| TAG(46:2) 12:0_16:1_18:1 | 0.354 ± 0.481 | 0.216 ± 0.262 | 0.193 | 0.433 |
| TAG(46:2) 14:0_16:1_16:1 | 0.171 ± 0.218 | 0.099 ± 0.127 | 0.144 | 0.415 |
| TAG(46:2) 14:1_16:0_16:1 | 0.128 ± 0.124 | 0.092 ± 0.107 | 0.262 | 0.489 |
| TAG(48:2) 12:0_18:1_18:1 | 0.165 ± 0.087 | 0.133 ± 0.071 | 0.143 | 0.415 |
| TAG(48:2) 14:0_16:0_18:2 | 0.389 ± 0.232 | 0.330 ± 0.256 | 0.375 | 0.561 |
| TAG(48:2) 14:0_16:1_18:1 | 0.258 ± 0.148 | 0.209 ± 0.146 | 0.221 | 0.460 |
| TAG(48:2) 14:1_16:1_18:0 | 0.115 ± 0.065 | 0.100 ± 0.078 | 0.462 | 0.630 |
| TAG(49:2) 15:0_16:1_18:1 | 0.219 ± 0.186 | 0.171 ± 0.169 | 0.325 | 0.531 |
| TAG(49:2) 16:0_16:1_17:1 | 0.150 ± 0.133 | 0.107 ± 0.106 | 0.199 | 0.433 |
| TAG(50:2) 14:0_18:1_18:1 | 0.135 ± 0.051 | 0.124 ± 0.055 | 0.448 | 0.621 |
| TAG(50:2) 16:0_16:1_18:1 | 0.263 ± 0.150 | 0.259 ± 0.197 | 0.923 | 0.965 |
| TAG(51:2) 16:0_17:1_18:1 | 0.227 ± 0.200 | 0.180 ± 0.185 | 0.367 | 0.561 |
| TAG(52:2) 16:0_18:0_18:2 | 0.089 ± 0.053 | 0.069 ± 0.031 | 0.091 | 0.359 |
| TAG(52:2) 16:0_18:1_18:1 | 0.924 ± 0.546 | 0.858 ± 0.643 | 0.687 | 0.823 |
| TAG(52:2) 16:1_18:1_18:0 | 0.112 ± 0.063 | 0.089 ± 0.040 | 0.118 | 0.415 |
| TAG(53:2) 17:0_16:0_20:2 | 0.116 ± 0.114 | 0.085 ± 0.092 | 0.263 | 0.489 |
| TAG(53:2) 17:0_18:1_18:1 | 0.723 ± 0.685 | 0.543 ± 0.577 | 0.296 | 0.506 |
| TAG(54:2) 16:0_18:1_20:1 | 0.178 ± 0.212 | 0.090 ± 0.125 | 0.067 | 0.314 |
| TAG(54:2) 18:0_18:1_18:1 | 0.964 ± 1.071 | 0.611 ± 0.516 | 0.127 | 0.415 |
| TAG(56:2) 16:0_20:1_20:1 | 0.101 ± 0.139 | 0.060 ± 0.060 | 0.162 | 0.415 |
| TAG(56:2) 18:0_18:1_20:1 | 0.264 ± 0.409 | 0.154 ± 0.206 | 0.216 | 0.454 |
| TAG(56:2) 18:1_18:1_20:0 | 0.210 ± 0.306 | 0.126 ± 0.132 | 0.192 | 0.433 |
| TAG(58:2) 18:0_18:2_22:0 | 0.062 ± 0.091 | 0.032 ± 0.027 | 0.106 | 0.398 |
| TAG(58:2) 18:1_18:1_22:0 | 0.321 ± 0.478 | 0.178 ± 0.147 | 0.142 | 0.415 |
| TAG(46:3) 12:0_16:1_18:2 | 0.487 ± 0.852 | 0.228 ± 0.285 | 0.139 | 0.415 |
| TAG(46:3) 14:0_14:1_18:2 | 0.848 ± 1.371 | 0.404 ± 0.555 | 0.124 | 0.415 |
| TAG(46:3) 14:1_14:1_18:1 | 0.682 ± 1.061 | 0.353 ± 0.530 | 0.154 | 0.415 |
| TAG(48:3) 12:0_18:1_18:2 | 1.194 ± 1.088 | 0.759 ± 0.594 | 0.072 | 0.321 |
| TAG(48:3) 14:0_16:0_18:3 | 0.513 ± 0.515 | 0.340 ± 0.316 | 0.141 | 0.415 |
| TAG(48:3) 14:0_16:1_18:2 | 2.007 ± 1.840 | 1.375 ± 1.317 | 0.150 | 0.415 |
| TAG(48:3) 14:1_16:1_18:1 | 2.015 ± 1.803 | 1.392 ± 1.348 | 0.154 | 0.415 |
| TAG(49:3) 15:0_16:1_18:2 | 0.406 ± 0.458 | 0.259 ± 0.289 | 0.163 | 0.415 |
| TAG(50:3) 14:0_18:1_18:2 | 2.933 ± 1.720 | 2.482 ± 2.037 | 0.379 | 0.561 |
| TAG(50:3) 14:1_18:1_18:1 | 0.364 ± 0.207 | 0.302 ± 0.202 | 0.270 | 0.491 |
| TAG(50:3) 16:0_16:0_18:3 | 0.330 ± 0.229 | 0.257 ± 0.177 | 0.198 | 0.433 |
| TAG(50:3) 16:0_16:1_18:2 | 1.767 ± 1.018 | 1.465 ± 1.021 | 0.276 | 0.491 |
| TAG(50:3) 16:1_16:1_18:1 | 2.932 ± 1.704 | 2.478 ± 2.010 | 0.370 | 0.561 |
| TAG(51:2) 16:1_17:0_18:1 | 0.503 ± 0.428 | 0.369 ± 0.338 | 0.202 | 0.433 |
| TAG(51:3) 15:0_18:1_18:2 | 0.673 ± 0.635 | 0.469 ± 0.448 | 0.177 | 0.425 |
| TAG(51:3) 16:0_17:1_18:2 | 0.545 ± 0.475 | 0.426 ± 0.485 | 0.359 | 0.559 |
| TAG(51:3) 16:1_17:0_18:2 | 0.307 ± 0.267 | 0.238 ± 0.293 | 0.370 | 0.561 |
| TAG(52:3) 16:0_18:0_18:3 | 0.117 ± 0.089 | 0.094 ± 0.075 | 0.314 | 0.525 |
| TAG(52:3) 16:0_18:1_18:2 | 2.487 ± 1.485 | 2.101 ± 1.172 | 0.291 | 0.504 |
| TAG(52:3) 16:1_18:1_18:1 | 4.725 ± 2.759 | 4.274 ± 2.755 | 0.547 | 0.705 |
| TAG(53:3) 17:0_18:1_18:2 | 0.548 ± 0.499 | 0.380 ± 0.336 | 0.149 | 0.415 |
| TAG(54:3) 18:0_18:1_18:2 | 0.400 ± 0.234 | 0.308 ± 0.134 | 0.081 | 0.351 |
| TAG(54:3) 18:1_18:1_18:1 | 6.674 ± 3.468 | 6.272 ± 4.039 | 0.693 | 0.828 |
| TAG(56:3) 18:1_18:1_20:1 | 1.116 ± 1.036 | 0.903 ± 1.037 | 0.450 | 0.621 |
| TAG(48:4) 12:0_18:1_18:3 | 0.525 ± 0.866 | 0.284 ± 0.404 | 0.193 | 0.433 |
| TAG(48:4) 12:0_18:2_18:2 | 0.692 ± 1.053 | 0.330 ± 0.441 | 0.105 | 0.398 |
| TAG(50:4) 14:0_18:1_18:3 | 0.851 ± 0.836 | 0.630 ± 0.708 | 0.297 | 0.506 |
| TAG(50:4) 16:1_16:1_18:2 | 0.971 ± 0.890 | 0.819 ± 1.110 | 0.577 | 0.733 |
| TAG(51:4) 15:0_18:2_18:2 | 0.370 ± 0.418 | 0.227 ± 0.258 | 0.134 | 0.415 |
| TAG(52:4) 14:0_18:1_20:3 | 0.098 ± 0.081 | 0.081 ± 0.082 | 0.430 | 0.608 |
| TAG(52:4) 16:0_16:0_20:4 | 0.181 ± 0.145 | 0.131 ± 0.092 | 0.130 | 0.415 |
| TAG(52:4) 16:0_18:2_18:2 | 2.144 ± 1.784 | 1.649 ± 1.187 | 0.233 | 0.469 |
| TAG(52:4) 16:1_18:0_18:3 | 1.322 ± 0.978 | 1.059 ± 0.837 | 0.289 | 0.504 |
| TAG(52:4) 16:1_18:1_18:2 | 1.026 ± 0.621 | 0.906 ± 0.703 | 0.504 | 0.669 |
| TAG(53:4) 17:0_18:1_18:3 | 0.406 ± 0.360 | 0.297 ± 0.299 | 0.230 | 0.466 |
| TAG(53:4) 17:0_18:2_18:2 | 0.376 ± 0.335 | 0.266 ± 0.266 | 0.184 | 0.433 |
| TAG(54:4) 16:0_18:0_20:4 | 0.157 ± 0.133 | 0.125 ± 0.096 | 0.309 | 0.521 |
| TAG(54:4) 16:0_18:1_20:3 | 0.222 ± 0.181 | 0.190 ± 0.183 | 0.510 | 0.670 |
| TAG(54:4) 18:0_18:1_18:3 | 0.116 ± 0.068 | 0.095 ± 0.052 | 0.208 | 0.439 |
| TAG(54:4) 18:0_18:2_18:2 | 0.212 ± 0.113 | 0.171 ± 0.078 | 0.126 | 0.415 |
| TAG(54:4) 18:1_18:1_18:2 | 2.631 ± 1.191 | 2.230 ± 0.795 | 0.150 | 0.415 |
| TAG(50:5) 14:0_18:2_18:3 | 0.284 ± 0.457 | 0.144 ± 0.198 | 0.149 | 0.415 |
| TAG(52:5) 16:0_16:1_20:4 | 1.063 ± 1.254 | 0.728 ± 0.862 | 0.255 | 0.487 |
| TAG(52:5) 16:0_18:2_18:3 | 1.607 ± 1.919 | 1.007 ± 1.023 | 0.156 | 0.415 |
| TAG(54:5) 16:0_18:1_20:4 | 1.622 ± 1.658 | 1.182 ± 1.148 | 0.259 | 0.489 |
| TAG(54:5) 16:1_18:1_20:3 | 0.168 ± 0.154 | 0.126 ± 0.117 | 0.258 | 0.489 |
| TAG(54:5) 18:0_18:2_18:3 | 0.776 ± 0.509 | 0.647 ± 0.468 | 0.333 | 0.537 |
| TAG(54:5) 18:1_18:2_18:2 | 1.896 ± 1.622 | 1.393 ± 0.866 | 0.159 | 0.415 |
| TAG(56:5) 18:0_18:1_20:4 | 0.386 ± 0.241 | 0.326 ± 0.195 | 0.315 | 0.525 |
| TAG(56:5) 18:0_18:2_20:3 | 0.129 ± 0.094 | 0.109 ± 0.078 | 0.394 | 0.577 |
| TAG(52:6) 14:0_16:0_22:6 | 0.291 ± 0.488 | 0.130 ± 0.176 | 0.113 | 0.412 |
| TAG(52:6) 16:1_18:2_18:3 | 0.292 ± 0.445 | 0.174 ± 0.270 | 0.244 | 0.476 |
| TAG(54:6) 16:0_16:0_22:6 | 0.173 ± 0.170 | 0.188 ± 0.236 | 0.792 | 0.879 |
| TAG(54:6) 16:0_18:1_20:5 | 0.145 ± 0.135 | 0.119 ± 0.103 | 0.417 | 0.598 |
| TAG(54:6) 16:0_18:2_20:4 | 1.297 ± 1.637 | 0.802 ± 0.871 | 0.170 | 0.421 |
| TAG(54:6) 18:1_18:2_18:3 | 0.944 ± 0.955 | 0.654 ± 0.677 | 0.200 | 0.433 |
| TAG(56:6) 16:0_18:1_22:5 | 0.834 ± 0.842 | 0.677 ± 0.680 | 0.450 | 0.621 |
| TAG(56:6) 18:0_18:2_20:4 | 0.773 ± 0.697 | 0.593 ± 0.468 | 0.267 | 0.491 |
| TAG(54:7) 16:0_16:1_22:6 | 0.636 ± 0.745 | 0.575 ± 0.944 | 0.791 | 0.879 |
| TAG(54:7) 16:0_18:2_20:5 | 0.233 ± 0.334 | 0.139 ± 0.171 | 0.198 | 0.433 |
| TAG(54:7) 16:1_18:2_20:4 | 0.205 ± 0.324 | 0.115 ± 0.156 | 0.195 | 0.433 |
| TAG(54:7) 18:2_18:2_18:3 | 0.519 ± 0.793 | 0.290 ± 0.423 | 0.189 | 0.433 |
| TAG(56:7) 16:0_18:1_22:6 | 0.296 ± 0.410 | 0.190 ± 0.232 | 0.246 | 0.477 |
| TAG(56:7) 16:1_18:1_22:5 | 0.579 ± 0.757 | 0.401 ± 0.496 | 0.310 | 0.521 |
| TAG(56:7) 18:0_18:2_20:5 | 0.106 ± 0.113 | 0.084 ± 0.068 | 0.379 | 0.561 |
| TAG(56:7) 18:1_18:2_20:4 | 0.522 ± 0.617 | 0.334 ± 0.307 | 0.160 | 0.415 |
| TAG(56:8) 18:1_18:2_20:5 | 0.112 ± 0.168 | 0.070 ± 0.078 | 0.241 | 0.476 |
| TAG(56:8) 18:2_18:2_20:4 | 0.121 ± 0.187 | 0.063 ± 0.076 | 0.141 | 0.415 |
| TAG(58:8) 18:1_18:1_22:6 | 0.221 ± 0.175 | 0.226 ± 0.255 | 0.930 | 0.965 |
| TAG(56:9) 18:2_18:2_20:5 | 0.036 ± 0.068 | 0.017 ± 0.024 | 0.169 | 0.421 |
| TAG(58:9) 18:1_18:2_22:6 | 0.341 ± 0.400 | 0.281 ± 0.408 | 0.586 | 0.740 |
| TAG(58:10) 18:2_18:2_22:6 | 0.213 ± 0.389 | 0.133 ± 0.258 | 0.378 | 0.561 |

Results are shown as mean ± SD. Statistically significantly different results are highlighted by bold font. q-values represent statistical significance following false discover rate (FDR) adjustment to correct for multiple testing.
